# Bi-glandular and persistent enterovirus infection and distinct changes of the pancreas in slowly progressive type 1 diabetes mellitus

**DOI:** 10.1038/s41598-023-33011-7

**Published:** 2023-04-28

**Authors:** Tomoyasu Fukui, Tetsuro Kobayashi, Erika Jimbo, Kaoru Aida, Akira Shimada, Yoichi Oikawa, Yasumichi Mori, Takeshi Fujii, Rikako Koyama, Kazuhiko Kobayashi, Akira Takeshita, Soroku Yagihashi

**Affiliations:** 1 Division of Immunology and Molecular Medicine, Okinaka Memorial Institute for Medical Research, Tokyo, Japan; 2 Division of Diabetes, Metabolism and Endocrinology, Department of Medicine, Showa University School of Medicine, Tokyo, Japan; 3 Department of Endocrinology and Metabolism, Toranomon Hospital, Tokyo, Japan; 4 Department of Diabetes and Endocrinology, Kanoiwa Hospital, Yamanashi, Japan; 5 Department of Endocrinology and Diabetes, Saitama Medical University, Saitama, Japan; 6 Department of Pathology, Toranomon Hospital, Tokyo, Japan; 7 Department of Gastroenterology, Toranomon Hospital, Tokyo, Japan; 8 Graduate School of Agricultural and Life Sciences, The University of Tokyo, Tokyo, Japan; 9 Department of Exploratory Medicine on Nature, Life, and Man, Toho University of Medicine, Chiba, Japan

**Keywords:** Type 1 diabetes, Infection

## Abstract

In slowly progressive type 1 diabetes mellitus (SPIDDM), the pancreas shows sustained islet inflammation, pancreatitis, pancreatic acinar cell metaplasia/dysplasia (ADM), and intraepithelial neoplasia (PanIN), a precancerous lesion. The mechanisms underlying these changes remain unclear. The presence of enterovirus (EV) encoded-capsid protein 1 (VP1) and -2A protease (2A^pro^) and the innate immune responses of the pancreas were studied using immunohistochemistry and in situ hybridization in 12 SPIDDM and 19 non-diabetic control pancreases. VP1, 2A^pro^, and EV-RNA were detected in islets and the exocrine pancreas in all SPIDDM pancreases. Innate immune receptor, melanoma differentiation-associated gene 5 (MDA5), and interferon (IFN)-beta1 were intensified in the islets of SPIDDM patients with short disease duration. However, expressions of MDA5 and IFN-beta1were suppressed in those with longer disease duration. CD3^+^ T cell infiltration was observed in the VP1- and insulin-positive islets (insulitis) and exocrine acinar cells. CD11c^+^ dendritic cells (DCs) in islets were scarce in long-term SPIDDM. This study showed the consistent presence of EV, suggesting an association with inflammatory changes in the endocrine and exocrine pancreas in SPIDDM. Suppressed expressions of MDA5 and IFN-beta1, as well as decreased numbers of DCs in the host cells, may contribute to persistent EV infection and induction of ADM/PanIN lesions, which may potentially provide a scaffold for pancreatic neoplasms.

## Introduction

Slowly progressive type 1 diabetes (SPIDDM)^1–4^, referred to as latent autoimmune diabetes in adults (LADA) when seen later in life^5,6^, is the most prevalent clinical subtype of type 1 diabetes mellitus. This pathology presents in various clinical phases, and it accounts for 8–10% of non-insulin-requiring diabetes cases in Japan, but obtaining pancreases from autopsy cases is difficult^7^. In terms of pathological entities and nomenclature, SPIDDM and LADA are different: SPIDDM does not exclude young-onset cases^7–9^, whereas LADA excludes young-onset cases^5,6^. Progressive β-cell failure, which is associated with an initial non-insulin-requiring state and an ultimate insulin-dependent state over several years, and persistence of islet cell autoantibodies, initially including islet cell antibodies (ICAs)^1–3^ and glutamic acid decarboxylase (GAD) autoantibodies^4,8^, are considered characteristic clinical indices of SPIDDM. In 2015, the Japan Diabetes Society established diagnostic criteria^9^.

We recently reported high frequencies of insulitis and major histocompatibility complex (MHC) class I hyper-expression of the islets, extensive pancreatic exocrine tissue inflammation, pancreatic ductal intraepithelial neoplasia (PanIN) with parenchymal atrophy, and pancreatic weight reduction in SPIDDM pancreases^10,11^. PanIN is defined as a microscopic (usually < 5 mm) papillary neoplastic lesion that is assumed to arise in acinar cells differentiating into acinar-to-ductal metaplasia (ADM); it occasionally develops to pancreatic ductal adenocarcinoma (PDAC) or intraductal papillary mucinous neoplasm (IPMN) in humans^12–14^. ADM loses the acinar marker (i.e., amylase) and exhibits ductal markers (i.e., cytokeratin 19), and it is often associated with exocrine pancreatic inflammation^12–14^. The resulting pathological features are represented by diffuse exocrine pancreatic inflammation^10^ and reduced pancreatic weight^11^. However, the mechanisms underlying these effects remain unclear. We recently found that enterovirus (EV) is closely associated with exocrine, as well as endocrine, tissue inflammation in fulminant type 1 diabetes mellitus (FT1DM)^15^.

The present study examined the presence of EV in islets and the exocrine pancreas by immunohistochemistry (IHC) and in situ hybridization (ISH) in the pancreases of SPIDDM patients and non-diabetic control subjects (non-DCs). Furthermore, innate immune responses, inflammatory changes of the islets and exocrine pancreas, T-cell insulitis, ADM/PanIN changes, and CXC chemokine ligand 10 (CXCL10) in relation to EV infection were studied in pancreases with various durations of SPIDDM.

## Results

### Presence of EV in islets and exocrine pancreases of SPIDDM and Non-DCs

#### VP1 protein detected by IHC

**Figure 1:**
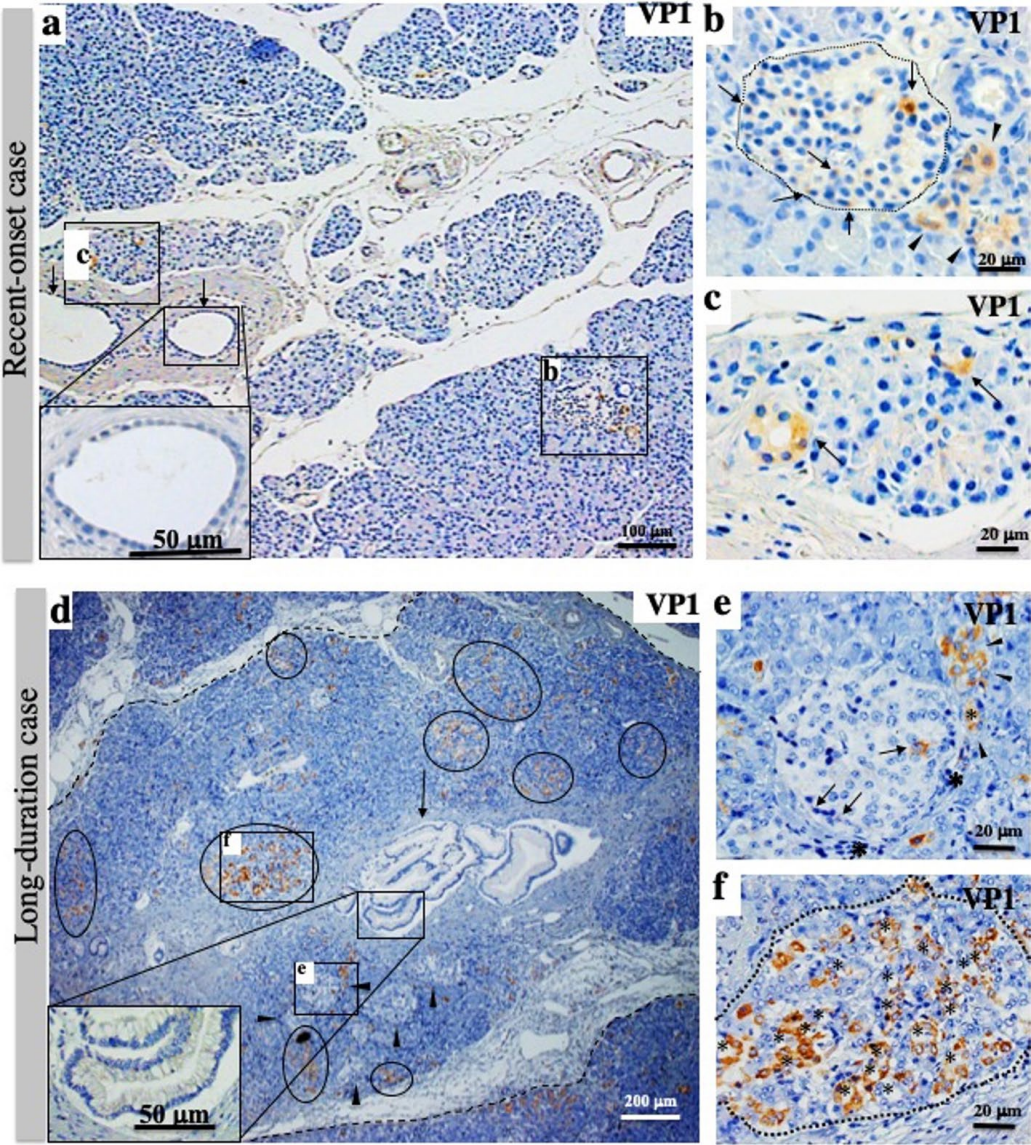
Enterovirus (EV) infection of islets and exocrine pancreas in recent-onset diabetes and long-duration diabetes of slowly progressive type 1 diabetes mellitus (SPIDDM). **(a)** Low-magnification view of the pancreas in recent-onset diabetes (Case SP10; duration: 3 months), stained for VP1. Islet cells (**b**) and exocrine cells (**c**) are positive for EV capsid protein (VP1, brown). VP1-positive acinar cells are rarely found. Inset: pancreatic duct epithelial cells (arrows) are intact, without features of pancreatic intraepithelial neoplasia (PanIN). (**b**) Magnified view of (**b**). Some islet cells (arrows) and acinar cells beside the islet (arrowheads) are positive for VP1. (**c**) Magnified view of (**c**). Two acini (arrows) show “ring-like” arrangements, reminiscent of the start of acinar-to-ductal-metaplasia (ADM). (**d)** Low-magnification view of the pancreas with a long duration of SPIDDM (Case SP8; duration: 14 years) stained for VP1. A PanIN (arrow and inset)-centered lobe demarcated by fibrous tissue (dashed lines) includes many islets, one of which is marked with (e), and VP1-positive exocrine acinar cells constitute clusters (circled), one of which is marked with (**f**). Note that parenchymal tissues in the PanIN lobe demarcated by a dashed line are atrophied due to fibrosis and contain a relatively increased number of islets (arrowheads). (**e**) Magnified view of (**e**) in (**d**). Islet cells are positive for VP1 (arrows) surrounded by infiltrated mononuclear cells (asterisks). Note that the VP1-positive acinar cell cluster (arrowhead) touches the islet, suggesting close association with the islet. (**f**) Magnified view of (**f**) in (**d**). Most exocrine VP1-positive acinar cells (brown, asterisks) show a flat shape with ring-like arrangements, a feature of ADM.

Staining for VP1 was positive in the islet cells of all 12 SPIDDM patients (Fig. 1a–c). In the recent-onset case (SP10; duration, 0.33 years), VP1 was observed in some islet cells and exocrine acinar cells (Fig. 1a–c). Unlike long-standing SPIDDM cases, small numbers of exocrine acinar cells were VP1-positive (Fig. 1a, c), and a proportion of VP1-positive acinar cells showed a small ring-like arrangement (Fig. 1c) and patchy degranulation of zymogen granules, a sign of beginning acinar-to-ductal metaplasia (ADM) (Supplemental Fig. S1a). In long-standing SPIDDM pancreases (duration, 3–24 years, n = 11), all pancreases contained VP1-positive islet cells and clustered VP1-positive ADM cells (Fig. 1d). Low-grade PanIN lesions, parenchymal cell inflammation, and lobular fibrosis were observed (Fig. 1d–f).

In non-DCs, islets were positive for VP1 in 5 of 19 (26%) cases with scattered ADM cells.

#### EV-RNA detected by ISH

**Figure 2:**
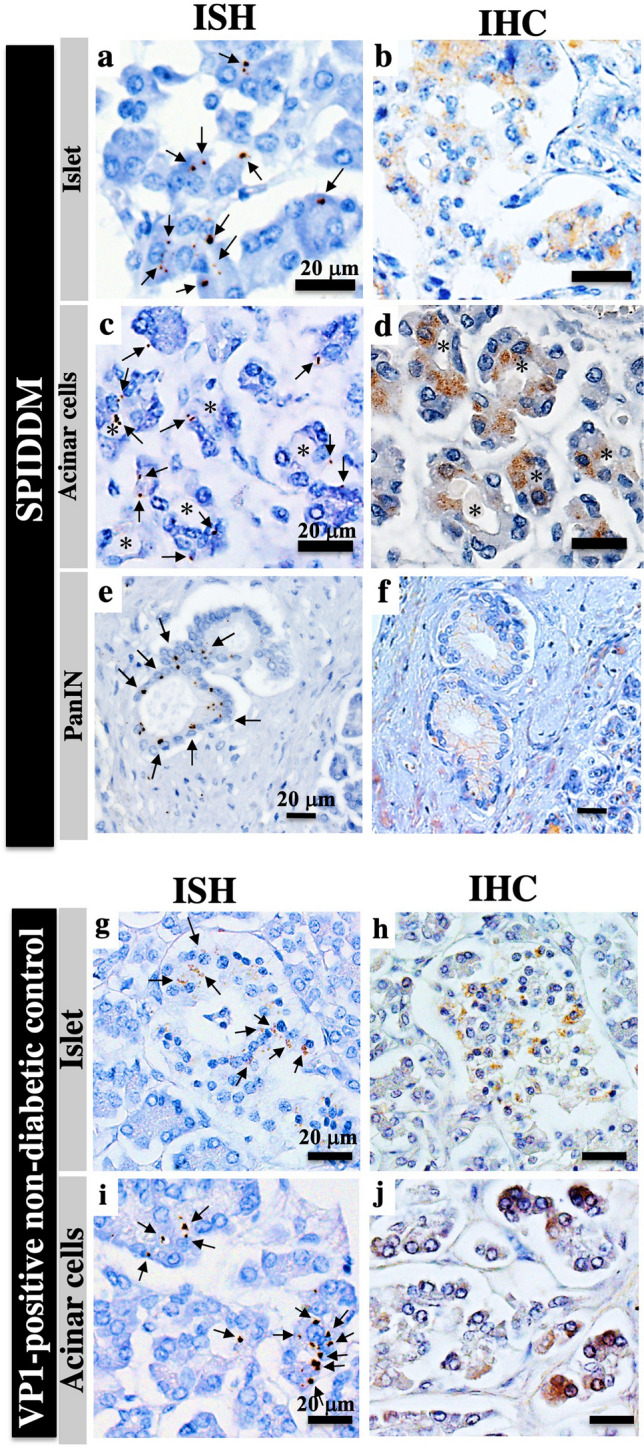
Detection of enterovirus (EV) probed for Coxsackievirus B1 (CVB1) RNA by in situ hybridization (ISH) and immunohistochemistry (IHC) in islets and exocrine tissues of SPIDDM and non-diabetic control (non-DC) pancreases. **(a)** Brown dots (arrows) are scattered in islet cells showing positive staining for CVB1 RNA by ISH in the islets of SPIDDM (Case SP2). Scale bar, 20 μm. (**b**) Serial section of (**a**) stained for VP1 in the islet by IHC (brown). (**c**) Brown dots are scattered, showing the presence of CVB1-RNA (arrows) in acinar cells changed to ADM (asterisks) of an SPIDDM pancreas (Case SP6). (**d**) Serial section of (**c**) stained for VP1 in acinar cells by IHC (brown). (**e**) Brown dots (arrows) show the presence of CVB1-RNA in a PanIN lesion in the SPIDDM pancreas (Case SP2). (**f**) Serial section of (**e**) stained for VP1 by IHC (brown) show VP1 protein residing in the same PanIN cells. (**g**) EV-RNA is detected by ISH (brown dots, arrows) in islet cells of a VP1-positive non-DC pancreas (Case non-DC17). (**h**) Serial section of (**g**) stained for VP1 in islet cells by IHC (brown). (**i**) EV-RNA (brown dots) is detected in acinar cells of a non-DC pancreas (arrows) (Case non-DC17). (**j**) Serial section of (**i**) stained for VP1 by IHC (brown). Same acinar cells as (**i**) are positive for VP1.

ISH demonstrated that EV-RNA for Coxsackie virus group B1 (CVB1), but not CVB3, was detected in 8 of 12 (67%) cases of islets (Fig. 2a, b) and in all 12 cases (100%) of exocrine pancreatic cells from SPIDDM pancreases (Fig. 2c–f). Eleven percent (2/19) and 16% (3/19) of non-DC pancreases were positive for EV-RNA in islets and exocrine pancreatic cells, respectively (Fig. 2g–j).

#### Frequencies of VP1-positive islets and VP1-positive exocrine pancreases in SPIDDM and non-DCs

**Figure 3:**
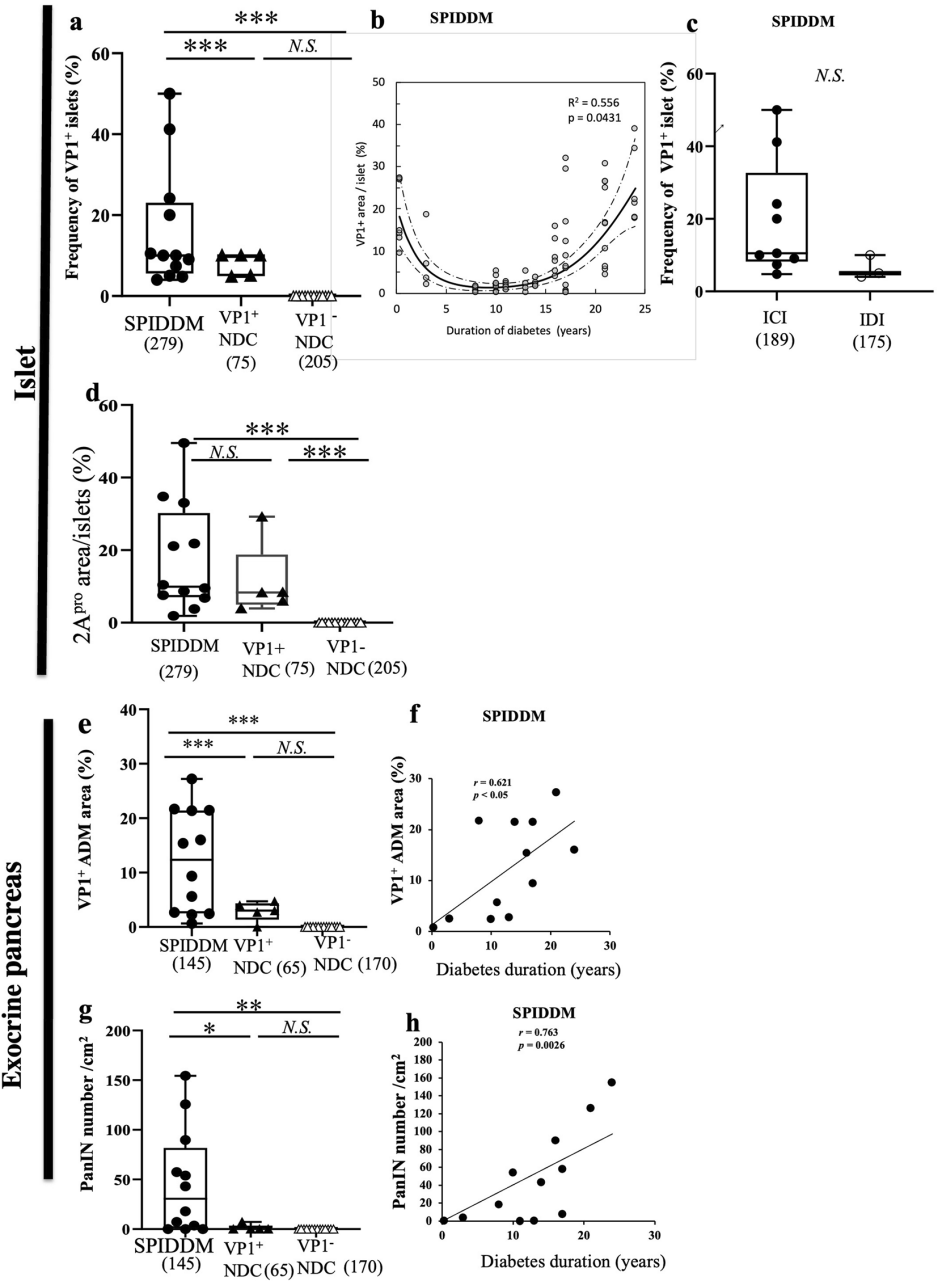
Quantified enterovirus (EV)-VP1 area in the islets, VP1-positive ADM cell area, and pancreatic intraepithelial neoplasia (PanIN) number in the exocrine pancreas of SPIDDM and non-diabetic control (non-DC) pancreas. **(a)** Overall frequencies of VP1-positive islets in SPIDDM and non-DC pancreases with medians depicted by box-and-whisker plots. The box represents the mid 50% of the data, and high and low whiskers represent the 95^th^ and 5^th^ percentiles, respectively. Open triangles show data for VP1-positive non-DC pancreases. Numbers in parentheses indicate numbers of islets counted. **P < 0.001 by the Mann–Whitney *U* test. (**b**) The islet VP1-positive area relative to the area of islets as related to the duration of diabetes in SPIDDM patients. The solid curve is a third-order polynomial function of the diabetes duration. A 95% confidence interval of the polynomial function was also constructed (dashed line). The fractional VP1-positive areas were cubic-root transformed to remove heterogeneity of the variance, which is significantly smaller in subjects with lower values of VP1-positive fractional area. The cube-root transform was also effective to remove the significant deviation of the fractional VP1-positive area from the normal distribution as judged by the Shapiro–Wilk test. Coefficients for the terms from 1st to 3rd orders were tested for statistical significance, and the p-value of the 3rd-order term is shown (*P* = 0.0431, n = 47). (**c**) Areas of VP1-positive islet area in insulin-containing islets (ICIs) and insulin-deficient islets (IDCs) in SPIDDM are insignificant. Open circles show cases completely devoid of residual beta cells. Numbers in parentheses indicate the number of islets counted. *N.S.* between two groups by the Mann–Whitney *U* test. (**d**) Areas of 2A^pro^-positive islet area in SPIDDM, VP1-positive non-diabetic controls (NDCs) were elevated but insignificant. Kruskal–Wallis test was used and numbers in parentheses indicate the number of islets counted. (**e**) Areas of VP1-positive ADM are significantly higher in SPIDDM pancreases than in VP1-positive non-DCs. Numbers in parentheses indicate the number of counted fields. ***P < 0.0001 by the Mann–Whitney *U* test. (**f**) VP1-positive ADM areas increase with increased duration of diabetes in SPIDDM. *P* < 0.05, Pearson’s correlation coefficient. (**g**) PanIN numbers per exocrine areas are significantly higher in SPIDDM than in non-DC. The number in parentheses indicates the number of counted fields. **P < 0.007, Mann–Whitney *U* test. (**h**) PanIN-positive lobe number in the exocrine pancreas increases with increased duration of diabetes in SPIDDM. *P* < 0.0026, Pearson’s correlation coefficient.

The median frequency of VP1-positive islets was 10.7%, higher than that of non-DCs (p < 0.0001, Fig. 3a). The fraction of VP-1 positive area showed U-shaped changes across the duration of diabetes characterized by sharp decline for the first 10 years and gradual increase thereafter (p < 0.0001, Fig. 3b). In addition, the frequencies of VP1 in insulin-containing islets (ICIs) were not significantly higher than those in islets devoid of beta cells (insulin-deficient islets) (Fig. 3c). EV-VP1 stained both islet beta cells and non-beta cells, suggesting a less-specific tropism of EV to islet cell subtypes (Supplemental Figs. S2b, c). High 2A^pro^-positive islets area were observed in all SPIDDM and 5 VP1-positive NDCs (Fig. 3d).

VP1-positive ADM was seen in all SPIDDM cases and 5 of 19 non-DCs (Fig. 3e). Exocrine VP1-positive ADM areas increased with longer duration of SPIDDM (Fig. 3f). The number of PanIN lesions was significantly greater in SPIDDM than in non-DC pancreases (Fig. 3g), and frequencies increased with increased duration of diabetes (Fig. 3h).

### Innate immune responses to EV in islets and exocrine pancreases of SPIDDM and non-DCs

#### Islets and exocrine pancreas in SPIDDM

**Figure 4:**
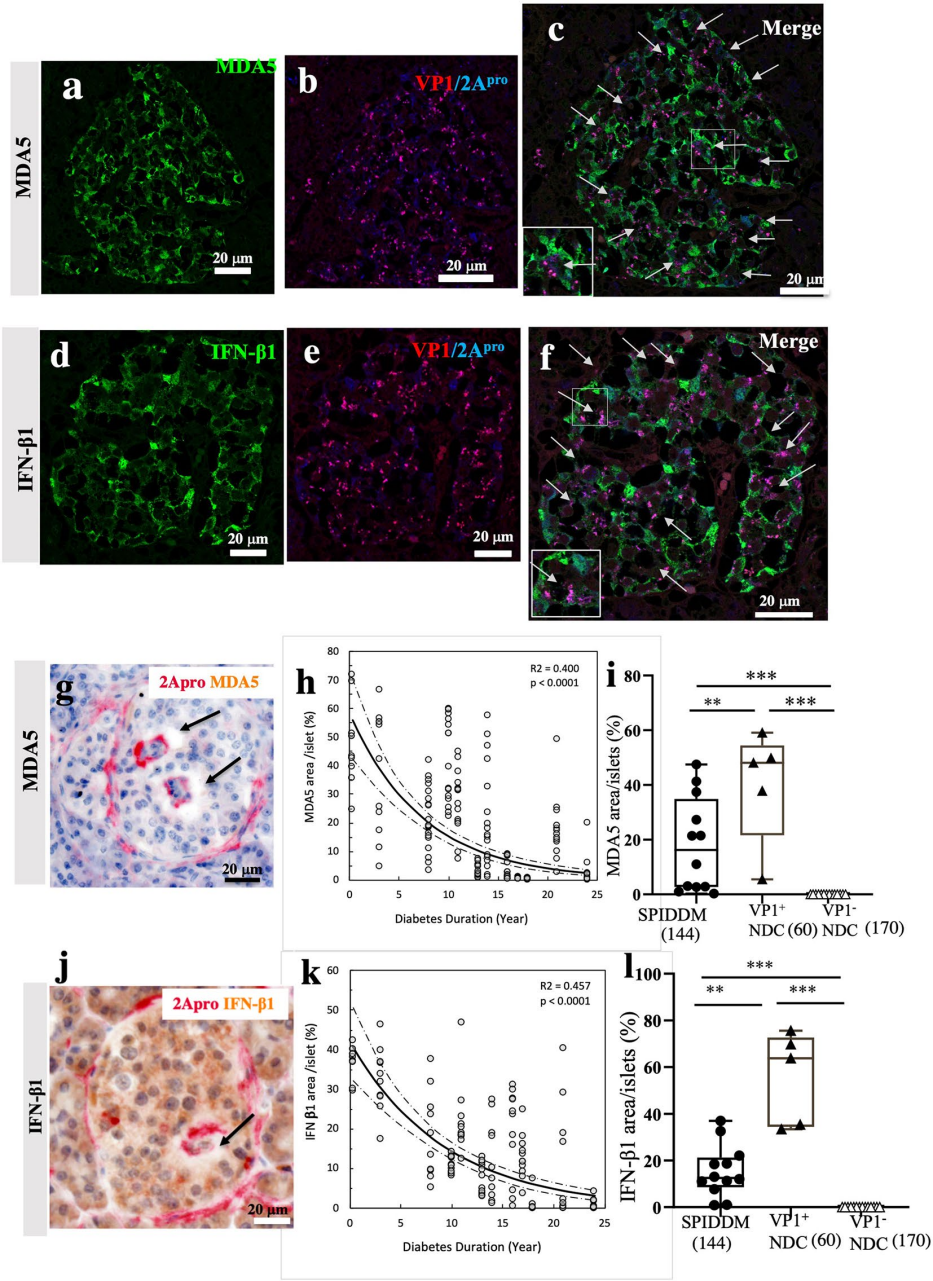
Innate immune responses to EV infection in the islets of SPIDDM pancreases. **(a–c)** MDA5 (**a**) and VP1/2A^pro^ (**b**) expression of SPIDDM islets. Merged image (**c**) shows MDA5 expression in the islets. The expression of MDA5 (brown) is lost around some cells, which express 2A^pro^ (red) as shown by the arrows. (**d–f**) IFN-β1 (**d**), VP1/2A^pro^ (**e**) expression of SPIDDM islets. Merged image (**f**) shows the expression of IFN-β1 (brown) is lost around some cells, which express 2A^pro^ as indicated by the arrows. (**g,j**) Double immunostaining for 2A^pro^ (red, **g,j**) and MDA5 (**g**, brown) or IFN-β1 (**j**, brown) show that where 2A^pro^ (red) was present in and around islet cells, MDA5 or IFN-β1 area was lost (arrows). (**h**) The MDA5-positive cell area relative to the islet area as related to the duration of diabetes (p < 0.0001, R^2^ = 0.393, n = 167). The solid curve depicts an exponential decay function of the diabetes duration (Y): AF_MDA5 = a EXP (b Y), where AF_MDA5 is the fractional area of MDA5-positive cells, which was square-root transformed to suppress heterogeneity of variances among the durations and also improve fitting to normal distribution for each duration. The coefficients were estimated at a = 57.9 (%) and b = −0.135 (p < 0.0001; 95% CI [−0.161, −0.109]). Also shown are 95% confidence boundaries (dashed curves) of the exponential function. (k) Temporal change of the IFN-β1-positive cell area relative to the islet area across the duration of diabetes (p < 0.0001, R^2^ = 0.446, n = 122). The solid curve depicts an exponential decay function of the diabetes duration (Y): AF_ IFN-β1 = a EXP (b Y), where AF_ IFN-β1 is the fractional area of IFN-β1-positive cells, which was square-root transformed to suppress heterogeneity of variances among the durations and improve fitting to normal distribution at each duration. The coefficients were estimated at a = 42.0 (%) and b = −0.108 (p < 0.0001; 95% CI [−0.129, −0.085]). Also shown are 95% confidence boundaries (dashed curves) of the exponential function. (**i,l**) Expression of MDA5 and IFN-β1 in the islets of SPIDDM was decreased compared with VP1-positive non-DC.

In all SPIDDM pancreases, MDA5 and IFN-β1 were expressed in islet cells positive for VP1 and 2A^pro^ (Fig. 4a–f). In addition, 2A^pro^ was detected in the same VP1-positive islet cells (Fig. 4e, f, Supplemental Fig. S3a–c). Interestingly, we found that where 2A^pro^ and/or VP1 was present in islet cells, no MDA5 or IFN-β1 was present (Fig. 4c, f, g, j, Supplementary Figs. S3a–c), named as blank space (BLS) (Fig. 4c, f, g, j, Supplemental Fig. S3a–c). No BLS was found in non-DCs. Furthermore, the islet area of MDA5-positive islet cells (Fig. 4h) and the islet area of IFN-β1-positive islet cells (Fig. 4k) decreased with increased duration of diabetes. The estimated decay rate for MDA5 was −0.135 with 95% CI of [−0.161, −0.109], p < 0.0001 and that for IFN-β1according was −0.108 with 95% CI of [−0.129, −0.085], p < 0.0001. The overlapping of CIs indicates no significant difference in the decay rate between MDA5 and IFN-β1, indicating both MDA5 and IFN-β1 disappeared at the same time statistically. These features may show that 2A^pro^ may biologically suppresses and/or deletes directly or indirectly MDA5 and IFN-β1 in situ^19,20^, contributing to decreasing islet areas of MDA5 and IFN-β1 during the course of the disease. The areas of MDA5 and IFN-β1 were significantly lower than those of non-DC (Fig. 4i, l).

No relationships were seen between MDA5 area or IFN-β1 area in islets and age of demise, age of onset, or residual beta cell volume (Supplemental Fig. S4a–f).

**Figure 5:**
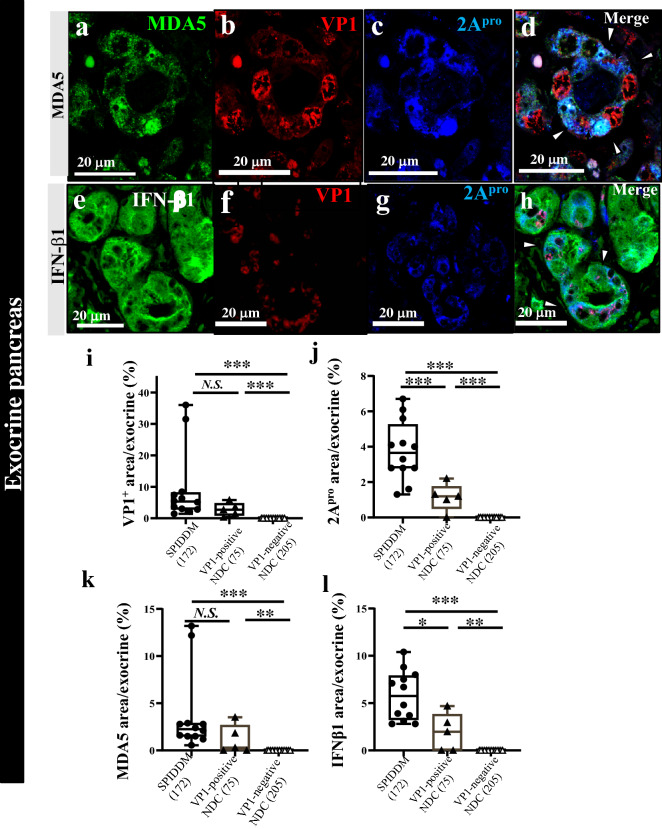
Innate immune responses to EV infection in the exocrine pancreas of SPIDDM and non-diabetic controls. **(a–d)** Triple immunostaining for MDA5 (**a**) VP1 (**b**), and 2A^pro^ (**c**) shows exocrine acinar cells changed to ADM in SPIDDM (Case SP7). Merged image (**d**) shows that VP1-positive (**b**, red) and 2A^pro^-positive cells (**c**, blue) show weak staining for MDA5 (**a**, green), in accordance with the concept that 2A^pro^ suppressed MDA5 expression in the same acinar cells. Scale bar, 20 μm. (**e–h**) Triple immunostaining for IFN-β1 (**e**), VP1 (**f**), and 2A^pro^ (**g**) of exocrine acinar cells changed to ADM. Merged image (**h**) of (**e–g**) shows that VP1 (red) is located in the perinuclear area and INF-β1 (blue, arrowheads) cytoplasm in EV-infected acinar cells. Scale bar, 20 μm. (**i,j**) VP1- and 2A^pro^-positive area in the exocrine pancreas of SPIDDM and VP1-positive non-diabetic control (NDC) are higher than those of VP1-negative NDC. Numbers in the parenthesis indicate counted exocrine area. One-way analysis of variance with Tukey analysis was used. (**k,l**) MDA5- and IFN-β1-positive area in the exocrine pancreas of SPIDDM and VP1-positive non-diabetic control (NDC) are higher than those of VP1-negative NDC. Numbers in the parenthesis indicate counted exocrine area. One-way analysis of variance with Tukey analysis was used.

MDA5, IFN-β1, VP1, and 2A^pro^ were also expressed in the exocrine pancreas of SPIDDM cases, who were positive for VP1 in the islet (Fig. 5a–l). In these cases, acinar cells changed to ADM (Fig. 5d, h).

#### Islets and exocrine pancreas in non-DCs

In five non-DCs, VP1 and/or EV-RNA, 2A^pro^, MDA5, and IFN-β1 were expressed (Fig. 2, Supplemental Fig. S5a–h) in the islets. In the 14 non-DCs negative for VP1 and/or EV-RNA, 2A^pro^, MDA5, and IFN-β1 were not expressed in exocrine cells (Fig. 5i–l). One VP1-positive non-DC (case non-DC17) showed positive VP1 in the centroacinar cells (Supplemental Fig. S6).

### VP1, inflammation, insulitis, and chemokine expression in islets and exocrine pancreases of SPIDDM and non-DCs

#### Inflammation and insulitis in islets of SPIDDM

**Figure 6:**
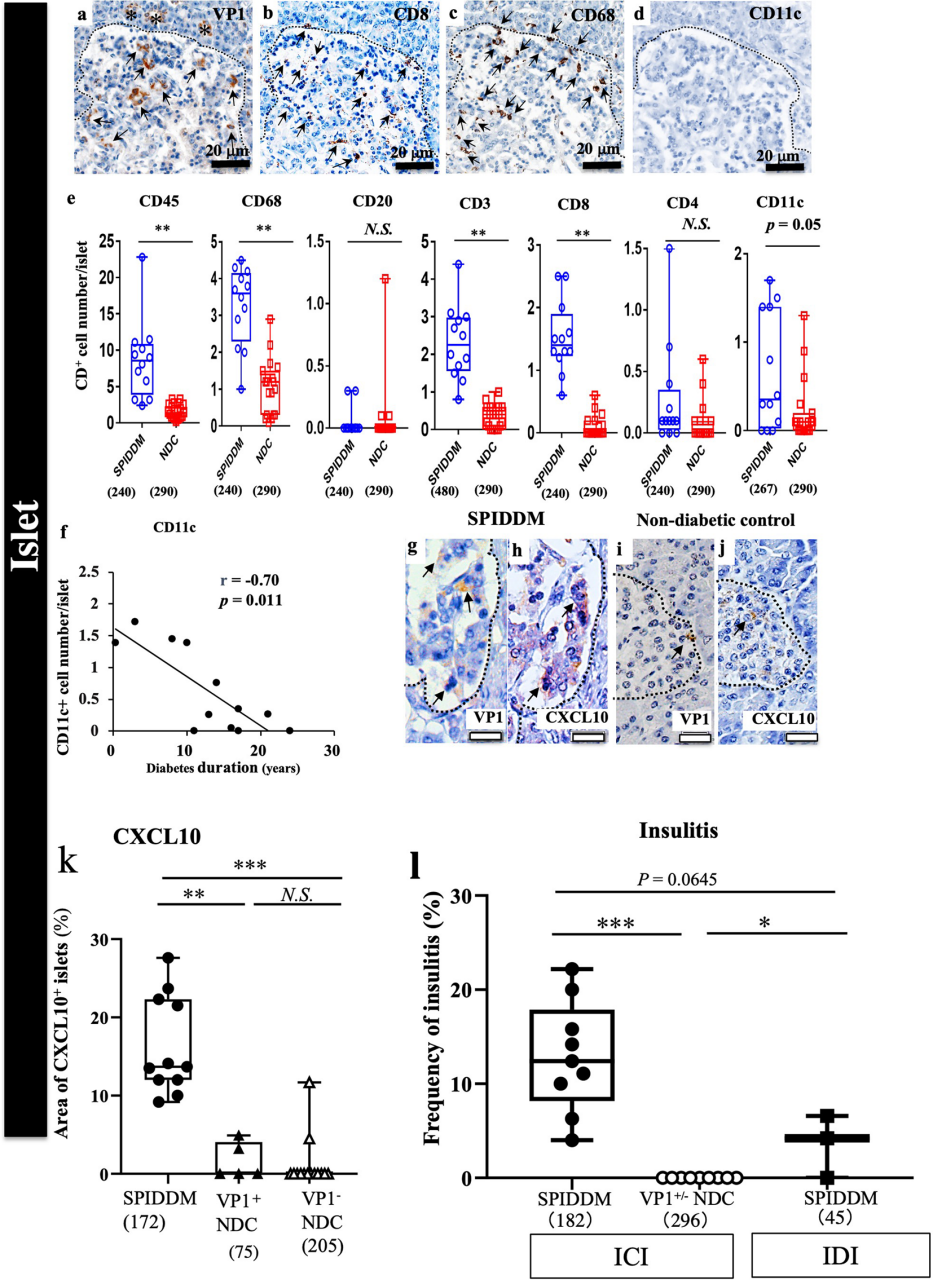
Characteristic features of EV infection and islet inflammation in SPIDDM. **(a–c)** Serial sections of islet stained for VP1 (**a**), CD8 (**b**), CD68 (**c**), and CD11c (**d**) in SPIDDM islets (Case SP2). The VP1-positive islet (**a**) is infiltrated by CD8^+^ T cells (**b**) and CD68^+^ macrophages (**c**). Scale bar: 20 μm. (**d**) Note that CD11c DC is not observed in the islet. (**e**) Numbers of CD45^+^, CD8^+^, and CD68^+^ cells, not CD20^+^, CD4^+^, and CD11c ^+^ cells, in islets are significantly higher in SPIDDM than in non-diabetic controls (NDCs). Numbers in parentheses indicate counted islets. **P < 0.01, Tukey’s test. (**f**) Number of CD11c^+^ DCs in islets decreases significantly with duration of SPIDDM. *P* < 0.01, Pearson’s correlation coefficient. (**g–j**) Consecutive sections stained for VP1 (**g,i**) and CXCL10 (**h,j**) in islets of SPIDDM and non-DC pancreases. VP1 (brown, arrows) is present in SPIDDM islet cells (**g**). (**h**) VP1-positive islet cells express CXCL10 in a high proportion of the SPIDDM pancreas. (**i,j**) In only 2 of 19 non-DC cases, both VP1 and CXCL10 are positive in islets (**i**) and CXCL10 (**j**). (**k**) CXCL10-positive area is significantly higher in islets in SPIDDM than in non-DCs. Numbers in parentheses indicate number of counted islets. **P < 0.001, Performed by Tukey’s test. (**l**) T-cell insulitis was observed in all cases of SPIDDM, whereas no T-cell insulitis was observed in VP1-positive or VP1-negative non-DCs (**l**). The frequencies of T-cell insulitis in insulin-containing islets (ICIs) and islets deficient beta cells (insulin-deficient islets: IDIs) in SPIDDM were insignificant. Performed by Tukey’s test.

VP1-positive islets were infiltrated by many CD subtypes of immune MNCs, mainly comprising CD68^+^ macrophages and CD8^+^ T cells (Fig. 6a–e). Importantly, the number of CD11c^+^ DCs in islets decreased significantly with the duration of SPIDDM (Fig. 6f).

#### CXCL10 expression in SPIDDM and non-DCs

A high proportion of VP1-positive islet cells expressed CXCL10 in all SPIDDM pancreases, whereas a small proportion of islet cells in non-DC pancreases expressed CXCL10 (Fig. 6g–k). Areas of CXCL10-expressing islet cells were significantly higher in SPIDDM islets than in non-DCs (Fig. 6k).

#### T-cell insulitis in SPIDDM

T-cell insulitis was observed in all cases of SPIDDM, whereas no T-cell insulitis was observed in VP1-positive or VP1-negative non-DCs (Fig. 6l). In addition, the frequencies of T-cell insulitis in insulin-containing islets (ICIs) were not significantly higher than those in islets deficient beta cells (insulin-deficient islets: IDIs) in SPIDDM.

#### Inflammation and changes in exocrine pancreas of SPIDDM and non-DCs

**Figure 7:**
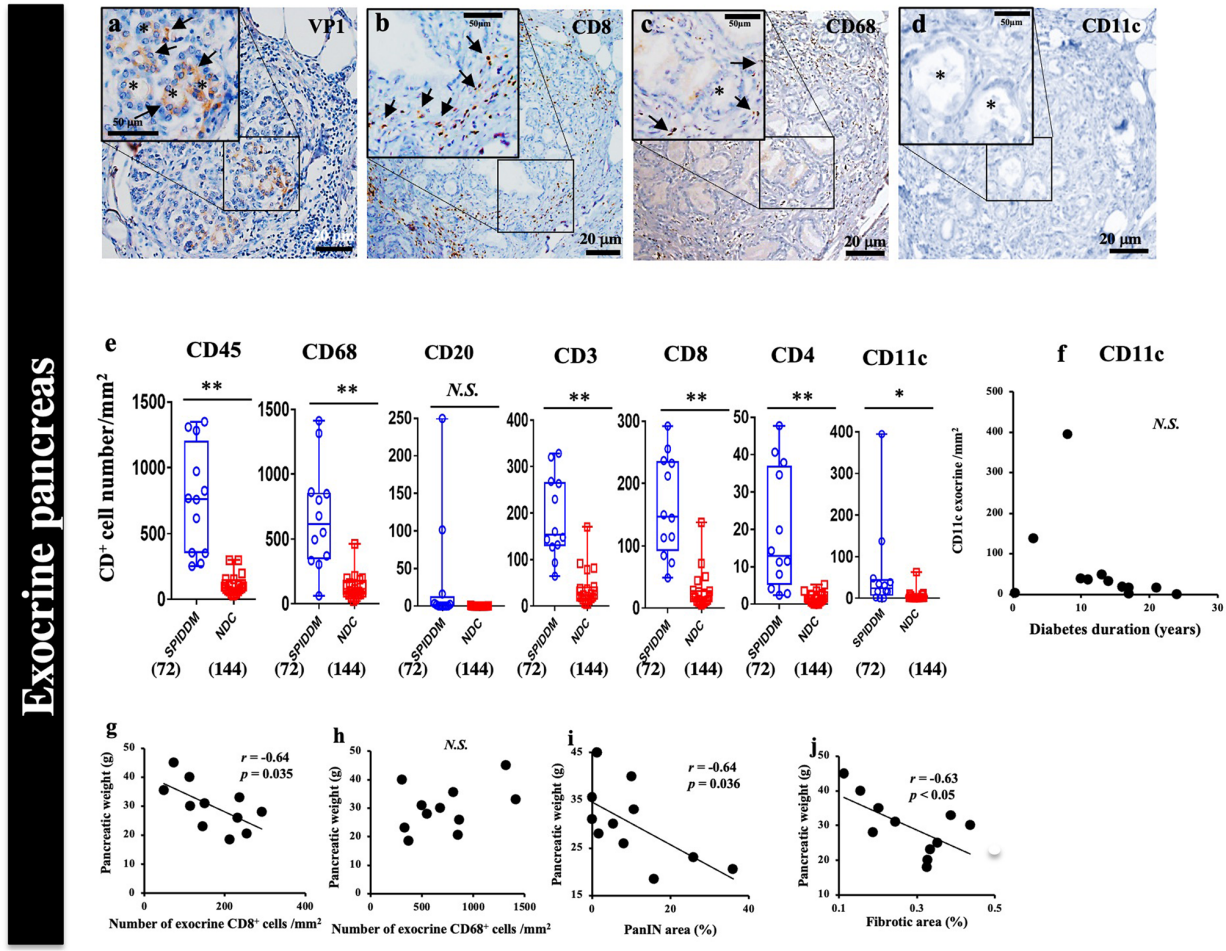
EV and pancreatic exocrine tissue inflammation and association with pancreatic weight in SPIDDM. **(a–d)** Serial sections of exocrine pancreas in a PanIN lobe stained for VP1 (**a**), CD8 (**b**), CD68 (**c**), and CD11c (**d**) in SPIDDM (Case SP2). VP1- and ADM-positive cell clusters (**a**, brown) are infiltrated by CD8^+^ T cells (**b**) and CD68^+^ macrophages (**c**). No CD11c^+^ cells are observed (**d**). Asterisks: ADM-changed acinar cells. (**e**) Numbers of CD45^+^, CD68^+^, CD8^+^, CD4^+^, and CD11c^+^ cells, but not CD20^+^ cells, in the exocrine pancreas are significantly higher in SPIDDM patients than in non-DCs (NDC). Numbers in parentheses indicate number of areas counted. *P < 0.05 and **P < 0.01, Tukey’s test. (**f**) The number of CD11c^+^ dendritic cells in the exocrine pancreas tends to decrease in some cases with duration of SPIDDM exceeding 10 years. (**g**) The number of CD8^+^ T cells in the exocrine pancreas of SPIDDM is negatively associated with SPIDDM pancreas weight. *P* = 0.035 by Pearson’s correlation coefficient. (**h**) The number of CD68^+^ macrophages in the exocrine pancreas of SPIDDM does not correlate with SPIDDM pancreas weight. (**i**) The PanIN area in SPIDDM pancreas correlates negatively with pancreatic weight. *P* = 0.036 by Pearson’s correlation coefficient. (**j**) The fibrosis area of SPIDDM correlates negatively with SPIDDM pancreas weight. *P* < 0.05 by Pearson’s correlation coefficient.

In PanIN lobes of exocrine pancreases in SPIDDM, most VP1-positive and ADM-positive acinar cells were infiltrated by CD3^+^ T cells, CD8^+^ T cells, and CD68^+^ macrophages (Fig. 7a–c, e). In non-PanIN lobes, inflammation was less severe (Supplemental Fig. S7a–p).

ADM changes were less frequent, and only one PanIN lesion was observed in 5 VP1-positive non-DCs (Fig. 3e). In one case of VP1-positive non-DCs, centroacinar cells were positive for VP1 (Fig, 3 g, Supplemental Fig. S6).

Since pancreatic weight was significantly decreased in SPIDDM^10,11^, relationships among exocrine inflammation, fibrosis, and pancreatic weight were studied. Numbers of CD8^+^ T cells, but not CD68^+^ macrophages, in pancreatic exocrine tissues correlated negatively with pancreatic weight (Fig. 7g, h). PanIN areas, in which exocrine inflammation and parenchymal fibrosis predominated, correlated negatively with parenchymal fibrotic area (Fig. 7i), and exocrine fibrotic area correlated negatively with pancreatic weight (Fig. 7j).

## Discussion

This is the first study of the pathogenesis of SPIDDM to show EV infection of both the endocrine and exocrine pancreas, and that EV infection leads to distinct pathological changes in both. In islet cells, EV infected and replicated accompanied by 2A^pro^ expression in islet cells, inducing chronic islet inflammation over time. In the exocrine pancreas, EV infection induced exocrine pancreatic inflammation, ADM, PanIN changes, and lobular fibrosis. In cases with short duration of SPIDDM, MDA5 and IFN-β1 were expressed in islet cells, as well as some exocrine acinar cells, suggesting critical innate immune activation against EV infection. MDA5 encoded by the *IFIH* gene, which influences the predisposition to type 1 diabetes mellitus^16^, is the main sensor of RNA viruses and inducer of type I IFN including IFN-β1, which prevents replication of viruses invading into host cells and is sometimes associated with autoimmune diseases^17^. Interestingly, expressions of MDA5 and IFN-β1 in islets were the highest in a recent-onset SPIDDM case and decreased with increased duration of diabetes (Fig. 4h, k). No significant difference of the decay rate between MDA5 and IFN-β1 was shown, indicating both MDA5 and IFN-β1 disappeared at the same time. One possibility may be the cleavage of MDA5 by EV-derived 2A^pro^ in persistent EV infection (Fig. 4a–f, Supplemental Fig S3a–c). In addition, 2A^pro^ plays a key role in inhibiting innate antiviral cellular responses, including inhibition of MDA5-initiated IFN-β promoter activation, mRNA expression, and downregulation of antiviral signaling cascades^18,19^. These results suggest the presence of multiple factors including 2A^pro^ and MDA5-IFN-β1 downregulating innate and adaptive immune pathways against EV infection, permitting persistent infection.

In addition, the numbers of CD11c^+^ DCs, primary initiators of antiviral adaptive immunity including Th1, Th2, and Th17 immune responses^20^, decreased with increased duration of diabetes in the islets in SPIDDM (Fig. 6f). Interestingly, CD11c^+^ DCs, which was assumed to express MDA5 in the cytosol, and EV infection alter the number and stimulation capacity of DCs, impairing the ability of the host to induce protective antiviral T-cell responses^21^. Moreover, EV infection in rodents causes a profound reduction in blood numbers of CD11c^+^ DCs^22^. Chronic EV infection of the pancreas may thus potentially suppress innate immunity through the MDA5–IFN-β1 pathway and CD11c^+^ DC-mediated EV-specific T-cell responses, permitting EV persistence and spread to the intact pancreas.

The frequencies of EV-positive islets decreased initially and later increased quadratically (Fig. 3b). The initial decrease of VP1-positive islets for up to 10 years after onset may be caused by intact innate and adaptive immunity against EV-infected islets. The later (over 10 years after onset) increase in the frequencies of VP1-positive islets may be related to suppressed host innate and adaptive immunities, including the MDA5–IFN-β1 pathway and DC-adaptive immune pathways, allowing the spread of EV to intact islets despite apparently activated CD8^+^ T cells and CD68^+^ macrophages in SPIDDM islets (Fig. 6a–c, e). Increased VP1 positivity in the duodenum over 10 years compared to 0–10 years among cases with type 1 diabetes mellitus was reported by the nPOD-Virus Group^23^. Chronic EV infection may activate pre-existing autoreactive T cells, non-beta cell-specific bystander T cells, and EV-specific T cells and induce islet cell neoantigens to trigger and accelerate islet autoimmunity^24,25^.

The frequencies of T-cell insulitis was insignificant between ICI and IDI in the cases of SPIDDM (Fig. 6l). Considering together with that frequency of VP1-positive islets between ICI and IDI are insignificant (Fig. 3c) in SPIDDM, beta cells and beta cell antigens are not always prerequisite but may play an important role on promote insulitis and islet autoimmunity in the catastrophic phase of islet inflammation^25^.

There are a few repots on EV infection and pathological changes of the exocrine pancreas^26–29^. The bi-glandular EV infection seen in the SPIDDM pancreas is unique. The present study showed that high proportions of acinar cells were infected by EV with the beginning ofADM changes in a recent-onset SPIDDM case (Fig. 1a, c). In cases with a long duration of SPIDDM, acinar cells were infected by EV and changed to ADM and PanIN lesions with chronic exocrine pancreatic inflammation composed of CD8^+^ T cells and CD68^+^ macrophages. ADM is driven by some intrinsic and extrinsic stressors, including viral infections and associated cytokines from infiltrating macrophages and T cells, chronic inflammation, and cell destruction over a prolonged period^30^. The co-localization of EV-VP1 and -2A^pro^ in acinar cells and islets (Fig. 4b, e, g, j, Supplemental Fig. S3a–c) supports our hypothesis that persistent EV infection and exposure of islet and exocrine acinar cells to 2A^pro^, a kind of serine protease, may induce islet cell damage, islet autoimmunity, chronic pancreatic inflammation with parenchymal fibrosis, and reduced pancreatic weight in SPIDDM. Many immune-pathogenetic mechanisms of the proteolytic and inflammatory 2A^pro^ on persistently EV-infected cardiomyocytes in dilated cardiomyopathy with autoimmunity have been reported^31^. Interestingly, ADM and PanIN lesions were observed in SPIDDM and VP1-positive non-DCs, suggesting a causative role of persistent EV infection in these changes (Figs. 3a, d, e, 5a–c, i, j). Experimental CVB4 infection in mice showed pancreatic inflammation and ADM changes of acinar cells, similar to the human SPIDDM pancreas^32^.

The mechanisms of EV-infected islets and the exocrine pancreas in 5 non-DCs who were positive for EV-VP1 and EV-RNA (EV-positive non-DCs) (Fig. 2g–j, Supplemental Fig. 5a–h) and remained in a non-aggressive state without inflammation, insulitis, or islet cell antibodies, are unclear. The four-fold higher area of IFN-β1 expression in the islets of the 5 EV-positive non-DCs compared to 12 SPIDDM (Fig. 4i, l) may have contributed to the early, rapid clearance of EV by IFN-β1 in islets. Early and rapid clearance of EV from the pancreas may be related to low expression of CXCL10 (Fig. 6k). In contrast, the expression and serum levels of CXCL10 were elevated (Fig. 5k) in SPIDDM^33^. Intact type I IFN including IFN-β1 expression in pancreatic cells may prevent vigorous EV infection and the development of EV-induced islet cell autoimmunity, cytokine and chemokine expression, and progressive islet cell destruction by migratory antiviral and autoreactive T cells^34^.

The present results in non-DCs suggest that EV infection in the pancreas may be a frequently and repetitively occurring misfortune inducing autoimmunity (i.e., autoantibody seroconversion) or abolition of autoimmunity and potential cumulative beta cell damage or recovery of beta cells in non-diabetic humans. There is a need to identify decisive environmental and genetic factors.

The common criterion of insulitis^35^ was not used because pancreases from the present subjects displayed a high number of CD68^+^ macrophages infiltrating the exocrine pancreas, potentially interfering with the determination of insulitis in SPIDDM.

A key limitation of the present study was the cross-sectional design, resulting in the collection of autopsied cases with various durations of diabetes. More detailed evaluation of the relationship among VP1, 2A^pro^, and beta cell damage awaits further study.

## Materials and methods

### Ethics

This study was approved by the Toranomon Hospital Human Research Ethics Committee (approval no. 948; date: 2017.5.23). Written, informed consent was obtained from the next of kin of the autopsied patients and from the one pancreas-biopsied patient. All research methods were performed in accordance with relevant guidelines and regulations.

### Characteristics of subjects

We have reported on the pathological changes of SPIDDM pancreas between 1982 and 2014^11,36^. In the present study, the pancreases of patients who meet the criteria of SPIDDM^9^ were collected as autopsy samples, with the exception of one case with tissue obtained by biopsy^37^ (Table 1). All autopsied pancreases were obtained freshly within 5 h after death. Patients with heavy alcohol use and inflammatory bowel disease were excluded. None of the cases with SPIDDM and non-diabetic controls was a smoker. One case (Case SP7) with SPIDDM had undergone curative surgical resection of the tail of the pancreas for PDAC 1 year before the patient died.

**Table 1:** Clinical characteristics of the subjects with slowly progressive type 1 diabetes (SPIDDM) and non-diabetic controls (non-DC).

Case subject	Age (years)	Sex	Duration of diabetes (years)	GADAb (U/mL)/ICA (JDF U)	Pancreatic weight (g)	C-peptide (ng/mL)	BMI (kg/m^2^)	Treatment for diabetes	Non-insulin requiring period (years)	Cause of death
SPIDDM										
SP-1	56	F	3	12.5/5	30.0	0.50	19.7	Insulin	1.5	Gastrointestinal bleeding
SP-2	58	F	21	3.4/nd	20.5	1.50	19.2	Insulin	9.0	Meningitis
SP-3	56	M	10	3.4/nd	23.0	1.80	20.2	Insulin	9.98	Gastric cancer
SP-4	42	M	17	4.2/nd	28.0	2.80	20.4	Insulin	12.0	Esophageal cancer
SP-5	43	M	13	4.6/nd	31.0	1.17	21.1	Insulin	10.0	Small intestinal cancer
SP-6*	68	F	8	398.0/10	45.0	0.02	18.2	Insulin	1.2	Chronic renal failure
SP-7*	75	M	11	12.8/5	35.5	0.14	21.2	Insulin	0.8	Esophageal cancer
SP-8	87	M	14	5.8/5	18.4	0.05	24.3	Insulin	1.0	Myocardial infarction
SP-9*	55	F	16	23.1/nd	33.0	0.10	19.1	Insulin	10.1	Chronic renal failure
SP-10	65	F	0.33	151/+^a^	24.9	9 µg/day^b^	26.0	Insulin	0.26	na^c^
SP-11	56	F	17	3.4/5	25.9	0.50	16.9	Insulin	5.8	Cerebral infarction, pneumonia
SP-12	77	F	24	3.5/5	40.0	0.60	17.2	Insulin	3.5	Acute respiratory distress
Mean ± SD	62 ± 14	M/F: 5/7	12.0 ± 6.9	52.1 ± 116.6/na	29.9 ± 8.2	0.82 ± 0.89	20.3 ± 2.7		5.4 ± 4.5	
Range	42–87		0.3–24	3.4–398.0/5–10	18.4–40.0	0.05–2.80	17.2–26.0		0.3–12.0	
Non-DC										
NonDC-1	70	F	–	< 1.5/< 5	78.0	–	nd	–	–	Pneumonia
NonDC-2	59	F	–	< 1.5/< 5	85.0	–	nd	–	–	Renal failure
NonDC-3	65	F	–	< 1.5/< 5	95.1	–	nd	–	–	Cerebral infarction
NonDC-4	63	M	–	< 1.5/< 5	63.1	–	nd	–	–	Acute myocardial infarction
NonDC-5	64	M	–	< 1.5/< 5	72.5	–	nd	–	–	Cronic renal failure
NonDC-6	64	M	–	< 1.5/< 5	80.0	–	22.0	–	–	Pneumonia, Bile dact cancer
NonDC-7	53	M	–	< 1.5/< 5	78.0	–	27.3	–	–	Bile duct cancer
NonDC-8	74	F	–	< 1.5/< 5	78.8	–	21.6	–	–	Duodenal gastrinoma
NonDC-9	82	M	–	< 1.5/< 5	nd	–	20.3	–	–	Cerebral infarction, Duodenal carcinoma
NonDC-10	69	M	–	< 1.5/< 5	nd	–	19.4	–	–	Restroperitoneal tumor
NonDC-11	66	F	–	< 1.5/< 5	nd	–	22.7	–	–	Colorectal cancer
NonDC-12	70	F	–	< 1.5/< 5	nd	–	16.4	–	–	Bile duct cancer
NonDC-13	67	F	–	< 1.5/< 5	nd	–	24.2	–	–	Pneumonia, Duodenal carcinoma
NonDC-14	63	M	–	< 1.5/< 5	nd	–	20.9	–	–	Duodenal carcinoma
NonDC-15	59	M	–	< 1.5/< 5	nd	–	23.7	–	–	Bile duct cancer
NonDC-16	67	F	–	< 1.5/< 5	nd	–	18.3	–	–	Colon cancer
NonDC-17	72	M	–	< 1.5/< 5	nd	–	nd	–	–	Cirrhosis, small intestinal bleeding
NonDC-18	69	F	–	< 1.5/< 5	74.0	–	23.8	–	–	Bile duct cancer
NonDC-19	60	F	–	< 1.5/< 5	88.2	–	nd	–	–	Acute gastric bleeding, Duodenal carcinoma
Mean ± SD	66 ± 6	M/F: 10/9	–	< 1.5/< 5	79.2 ± 8.8	–	21.7 ± 2.9	–	–	
Range	53–82				63.1–95.1		16.4–27.3			

*The beta cell is completely abolished case.

^a^Positive for IA-2Ab.

^b^24-h urine C-peptide (normal 16–120 µg/day).

^c^Biopsied sample.

*nd* not detected, *na* not applicable.

### Tissue preparation and IHC

The tail and body of each pancreas were obtained for pathological analyses of islet morphology, pancreatic ductal changes, and parenchymal changes. Two blocks were obtained from each part. Pancreatic tissues were fixed in 4% formalin and embedded in paraffin. For quantification analyses, including β cell area, insulitis frequencies, and subtypes of mononuclear cells (MNCs) were determined in the islets and exocrine pancreatic tissues from the midpoint of the pancreas to the position one-quarter of the distance to the end of the tail of the pancreas, a portion of the pancreas in which the morphometric parameters are thought to be fairly representative of the entire organ^38^.

To minimize possible sampling bias for counting MNCs positive for CD45, CD68, CD3, CD8, CD4, CD20, and CD11c markers in the islets and exocrine pancreas, these cells were stained on serial sections (4-µm-thick) by immuno-peroxidase staining for each CD marker (Supplemental Table 1). Next, each section already stained for each CD marker was co-stained by immunofluorescence methods for insulin and glucagon to identify the exact location of the islet.

### Assessment of EV infection and inflammation in the endocrine and exocrine pancreases of SPIDDM

#### Optimization of EV-capsid protein 1 (VP1) and EV-encoded 2A protease (2A^pro^) staining by IHC

To establish the validity of IHC for VP1, the following preliminary experiments were conducted. First, in addition to antibody 5D8/1 (Dako, Ely, UK) (dilutions 1:200, 1:400, 1:800, and 1:1600), antibody 6E9-2 (generated using CVB5 as an immunogen) reacts with most EV strains of the echo, Coxsackie and poliovirus groups (Creative Diagnostics, NY, NY, USA) (1: 100) (kindly donated by Professor Antonio Toniolo, University of Insubria, Varese, Italy) was used to confirm the presence of VP1 immunoreactivity in serial pancreatic sections of SPIDDM.

Dilution of 5D8/1 antibody at 1:400 gave the lowest background color and reproducibility. Next, whether 5D8/1 (1:400) and 6E9-2 (1:100) antisera were absorbed with VP1 protein (Antibodies-online.com, Aachen, Germany) at 10 μg/ml was examined. The 5D8/1 and 6E9-2 antibodies when incubated overnight with VP1 protein showed complete abolition of immunostaining for VP1 (Supplemental Fig. S8a–h)^39,40^. Ten consecutive pancreatic sections in each case from 12 SPIDDM pancreases were stained for VP1 using 5D8/1 and 6E9-2. The concordance of islet staining results (positive using 6E9-2/positive using 5D8/1) and ADM-changed acinar cell staining results (positive using 6E9-2/positive using 5D8/1) was estimated at 120/120 [95% confidence interval (CI): 96.0–100%] and 117/120 (95% CI: 92.9–99.1%), respectively. The fraction of stained cells showed no significant difference between the antibodies (Fisher’s exact test, *P* = 0.247). From these results, it can be judged that 5D8/1 and 6E9-2 are equally efficient detectors of VP1 in islets and ADM-changed exocrine cells of SPIDDM.

As positive and negative controls for VP1 (5D8/1) by IHC, cultured Vero cells (green monkey kidney cells) that were infected or not infected by CVB1 (strain number: 16-20289), which was isolated from a Japanese child with aseptic meningitis in 2016, were used^40^. Vero cells at 0.1 and 3.0 multiplicity of infection (MOI)-infected and incubated for 24 h after infection and fixed by 5% formaldehyde were stained positive for VP1^40^. Mock cultured cells did not show positive staining for VP1^40^. As another positive control for VP1 antisera (5D8/1) by IHC, formalin-fixed and paraffin-embedded pancreas from FT1DM, which was ascertained by in situ hybridization^41^, was stained for VP1 (5D8/1) and was positive for enterovirus. New antisera against EV-2A^pro^ (ET2112) were raised, and it was confirmed that the antiserum stained CVB1 in virus-infected Vero cells and also stained VP1-positive islet cells of FT1DM^40,41^. In this study, this antiserum was absorbed with 2A^pro^ peptide of CVB1 at 10 ng/ml in SPIDDM (Supplemental Fig. S8i–l). As negative control, VP1-negative non-DC pancreas (Case non-DC 6) was used (Supplemental Fig. S9a–d). Based on the above preliminary results, antibody against 5D8/1 (1:400) for VP1 and antiserum against 2A^pro^ (ET2112) (1:6400) for 2A^pro^ were used.

#### Assessment of EV-RNA by in situ hybridization (ISH)

To detect EV-RNA by ISH, specific probes for CVB1 and CVB3 (RNA Scope 2.5 HD Detection Kit Brown; ACD, Newark, CA, USA) were used according to the protocol provided by the manufacturer. Negative (DapB) and positive (His-PPIB) control probes were included. Specific RNA staining was identified as punctate dots (Supplementary Fig S10a–f)^41^.

#### Assessment of islets positive for VP1 and islet inflammation

In principle, images of more than 15 islets with diameters exceeding 25 µm and more than ten alpha cells per pancreas were analyzed at 200× magnification by conventional microscopy (DP73; Olympus, Tokyo, Japan) and confocal laser scanning microscopy (FV3000; Olympus unless otherwise noted). VP1-positive islets were quantified by cellSens Dimension software (Olympus, Japan version 1.16). Two investigators evaluated cells independently in a double-blinded manner. T-cell insulitis was defined as a minimum of 3 islets with a threshold of ≥ 6 CD3^+^ cells within the islet plus immediately adjacent to the islet per pancreas section^42^, and T-cell insulitis was evaluated with the same numbers, as much as possible, per case in SPIDDM and non-DC pancreases.

#### Assessment of areas of 2A^pro^, innate immune markers, and chemokines/cytokines in islets and exocrine pancreas

Expressions of 2A^pro^, MDA5, IFN-β1, and CXCL10 in islets were examined using antisera (Supplemental Table 1), as previously described^40,43,44^. All images were quantified using cellSens Dimension software (version 1.16; Olympus), and the values are expressed as the percentages of the positive islet areas for 2A^pro^, MDA5, IFN-β1, or CXCL10 divided by the respective islet or exocrine pancreatic area. Exocrine pancreas was examined for VP1, MDA5,IFN-β1, and 2A^pro^.

#### Assessment of exocrine pancreatic inflammation, beta cell weight, and parenchymal fibrosis

Numbers of immune cells positive for CD markers among pancreatic exocrine tissues were counted in 5 randomly selected photos of pancreatic sections (1.4 × 1.1 mm^2^) in each case stained for CD markers and AZAN trichrome-stained fibrotic areas in exocrine pancreases at  100× magnification using cellSens Dimension software (version 1.16; Olympus). A total of 186 images were taken in SPIDDM (n = 72) and non-DC pancreases (n = 114). After quantification of images by computer software, all images and values analyzed by the software (cellSens Dimension software, version 1.16; Olympus) were reconfirmed on accurately acquired captured images by the two investigators and adjusted to ensure that counts were correct. Beta cell weights in SPIDDM cases had been estimated previously^11^.

#### Assessment of ADM and PanIN

ADM was defined as flat ductal-like acinar cells arranged like a ring, which had lost zymogen granules on hematoxylin and eosin (HE) staining. Prior to this observation, sections were stained for amylase and cytokeratin 19 (CK19), and 96% (288/300) of the ring-like-arranged acinar cells were confirmed as positive for CK19 and mostly devoid of amylase granules (Supplementary Fig S1a–c). The mean (± standard deviation [SD]) long diameter of ADM was 38 ± 13 µm (n = 67). Detection criteria for ADM were: (1) flat, ductal-like acinar cells arranged like a ring; and (2) long diameter of acini within the range of 12–64 µm (mean ± 2SD). Since most ADM-changed acinar cells were VP1-positive (as mentioned in the “Results”), quantification followed by segmentation of VP1-positive ADM cell cluster areas (VP1-positive ADM areas) was expressed as the percentage of the image captured using cellSens Dimension software (version 1.16; Olympus). Segmented images of VP1-positive ADM showed that cluster areas were tightly outlined and well segmented (Supplemental Fig. S11a, b). Positivity and segmentation of ADM cells were reconfirmed by two investigators.

PanIN was defined as ductal-like cells with tall columnar-shaped cytoplasm with enlargement of thick nuclei rich in chromatin. PanIN lobes were defined as easily recognized and counted as lobes surrounded by fibrous tissues with a central PanIN-positive pancreatic duct. The number of PanIN lesions was counted manually in 5 randomly selected photos of the pancreatic section (1.4 × 1.1 mm^2^) for each case at × 100 magnification. Non-PanIN lobes were defined as pancreatic lobes without PanIN lesions.

### Assay methods for GADAb and ICAs

GADAb levels were measured using a radioimmunoassay (RIA) method with a cut-off value of 1.5 U/mL^45^. ICAs were assayed by immunofluorescence methods (detection limit < 5 Juvenile Diabetes Foundation U, precision score 0.65, specificity 100%)^46^.

### Statistical analyses

Statistical analysis was performed using JMP Pro version 16.0.0 (SAS Institute, Cary, NC, USA).

For the analysis of the relationships between the diabetes duration and the areas of VP1, MDA5 and IFN-β1 per islets, polynomial and exponential functions were used to regression after correcting for heterogeneity of variance and non-normality of the error term as needed.

### Permit statement

Permission to reuse the data of our previously published paper was obtained from the publisher as follows: License No. 5359651426969. License data Jul 31, 2022. Licensed content publisher: Wolters Kluwer. Licensed content publication: Health, Inc. Pancreas 47, Agreement (PDF) is enclosed. Licensed Content Title: Distinct inflammatory changes of the pancreas of slowly progressive insulin-dependent-(Type 1) diabetes. Authors: Kaoru Aida, Tomoyasu Fukui, Erika Jimbo, et al. Volume: 47. Portions: Pancreas 47, page 11101–1109. Figures 1C,D and 3H. Creative Commons License: CC-BY-NC.

## Supplementary Information

Supplementary Table 1.

Supplementary Figures.

## Data Availability

The data presented in this manuscript are available on reasonable request to the corresponding author.
