# Supplementary material for: Bi-glandular and persistent enterovirus infection and distinct changes of the pancreas in slowly progressive type 1 diabetes mellitus
Source: Sci Rep. 2023 Apr 28;13:6977. doi: 10.1038/s41598-023-33011-7 (PMC10147722; doi:10.1038/s41598-023-33011-7)
Supplement: Supplementary file 1 — Supplementary Table 1. [file 41598_2023_33011_MOESM1_ESM.docx]

**Supplemental Table S1. Antibodies and dilution used in the immunohistochemical study.**

| **Antigen** | **Species** | **Clone** | **Source** | **Code** | **Dilution** |
| --- | --- | --- | --- | --- | --- |
| **Amylase** | **Goat** | **C-20** | **Santa Cruz, Dallas, TX** | **sc-128121** | **1:100** |
| **Insulin** | **Guinea pig** | **-** | **Dako, Carpinteria, CA** | **IR002** | **1:2** |
| **Glucagon** | **Rabbit** | **-** | **Bioss, Woburn, MA** | **bs-3796R** | **1:400** |
| **Enterovirus VP1 peptide** | **Mouse** | **5D8/1** | **Novocastra, Newcastle Upon Tyne, UK** | **NCL-ENTERO** | **1:400** |
| **Enterovirus VP1 peptide** | **Mouse** | **6E9-2** | **Creative Diagnostics** | **DMAB4995** | **1:100** |
| **Enterovirus 2A^pro^ peptide** | **Rabbit** | **-** | **House made (Biology)** | **ET2112** | **1:1600** |
| **CXCL10** | **Goat** | **-** | **R&D Systems, Minneapolis, MN** | **AF-266** | **1:80** |
| **CK19** | **Mouse** | **b170** | **Novocastra, Newcastle Upon Tyne, UK** | **NCL-CK19** | **1:150** |
| **CD45** | **Mouse** | **2B11 + PD7/26** | **Dako, Carpinteria, CA** | **IR751** | **1:2** |
| **CD3** | **Mouse** | **PS1** | **Novocastra, Newcastle Upon Tyne, UK** | **NCL-L-CD3-PS1** | **1:50** |
| **CD4** | **Mouse** | **1F6** | **Novocastra, Newcastle Upon Tyne, UK** | **NCL-CD4-1F6** | **1:50** |
| **CD8** | **Mouse** | **C8/144B** | **Dako, Carpinteria, CA** | **M7103** | **1:50** |
| **CD20** | **Mouse** | **L26** | **Dako, Carpinteria, CA** | **M0755** | **1:200** |
| **CD11c** | **Rabbit** | **EP1347Y** | **Bioss, Woburn, MA** | **bs-2508R** | **1:100** |
| **CD68** | **Mouse** | **PG-M1** | **Dako, Carpinteria, CA** | **M0876** | **1:50** |
| **IFN-β1** | **Rabbit** | **-** | **LSBio, Seattle, WA** | **LS-B1361** | **1:100** |
| **IFN-β1** | **Sheep** | **-** | **PBL,Assay Science** | **31401-1** | **1:50** |
| **MDA5** | **Goat** | **-** | **Abcam, Cambridge, UK** | **ab4544** | **1:50** |

| **Antigen** | **Name** | **Species** | **Source** | **Code** | **Dilution** |
| --- | --- | --- | --- | --- | --- |
| **Amylase** | **Anti-goat IgG Rhodamin-Red-X** | **Donkey** | **Jackson Immuno Research, Grove, PA** | **705-296-147** | **1:200** |
| **Insulin** | **Anti-guinea pig IgG Alexa Fluor 488** | **Donkey** | **Jackson Immuno Research, Grove, PA** | **706-545-148** | **1:400** |
| **Glucagon** | **Anti-rabbit IgG Alexa Fluor 594** | **Donkey** | **Jackson Immuno Research, Grove, PA** | **711-585-152** | **1:400** |
| **Enterovirus VP1 peptide** | **Anti-rabbit /mouse /HRP (Envision kit)** | **Goat** | **Dako, Carpinteria, CA** | **K5007** |  |
| **Enterovirus VP1 peptide** | **Anti-mouse IgG Alexa Fluor 488** | **Donkey** | **Jackson Immuno Research, Grove, PA** | **715-546-150** | **1:200** |
| **Enterovirus 2A^pro^ peptide** | **Anti-rabbit /mouse /HRP (Envision kit)** | **Goat** | **Dako, Carpinteria, CA** | **K5007** |  |
| **Enterovirus 2A^pro^ peptide** | **Anti-rabbit IgG AMCA** | **Donkey** | **Jackson Immuno Research, Grove, PA** | **711-156-152** | **1:50** |
| **Enterovirus 2A^pro^ peptide** | **Anti-rabbit IgG Alkaline Phosphatase** | **Donkey** | **ROCKLAND** | **611-705-127** | **1:400** |
| **CXCL10** | **Anti-goat IgG /HRP** | **Donkey** | **Jackson Immuno Research, Grove, PA** | **705-036-147** | **1:500** |
| **CK19** | **Anti-mouse IgG Alexa Fluor 488** | **Donkey** | **Jackson Immuno Research, Grove, PA** | **715-546-150** | **1:200** |
| **CD45** | **Anti-rabbit /mouse /HRP (Envision kit)** | **Goat** | **Dako, Carpinteria, CA** | **K5007** | **-** |
| **CD3** | **Anti-rabbit /mouse /HRP (Envision kit)** | **Goat** | **Dako, Carpinteria, CA** | **K5007** | **-** |
| **CD4** | **Anti-rabbit /mouse /HRP (Envision kit)** | **Goat** | **Dako, Carpinteria, CA** | **K5007** | **-** |
| **CD8** | **Anti-rabbit /mouse /HRP (Envision kit)** | **Goat** | **Dako, Carpinteria, CA** | **K5007** | **-** |
| **CD20** | **Anti-rabbit /mouse /HRP (Envision kit)** | **Goat** | **Dako, Carpinteria, CA** | **K5007** | **-** |
| **CD11c** | **Anti-rabbit /mouse /HRP (Envision kit)** | **Goat** | **Dako, Carpinteria, CA** | **K5007** | **-** |
| **CD68** | **Anti-rabbit /mouse /HRP (Envision kit)** | **Goat** | **Dako, Carpinteria, CA** | **K5007** | **-** |
| **IFN-β1** | **Anti-rabbit /mouse /HRP (Envision kit)** | **Goat** | **Dako, Carpinteria, CA** | **K5007** | **-** |
| **IFN-β1** | **Anti-sheep IgG FITC** | **Donkey** | **Jackson Immuno Research, Grove, PA** | **713-096-147** | **1:50** |
| **MDA5** | **Anti-goat IgG /HRP** | **Donkey** | **Jackson Immuno Research, Grove, PA** | **705-036-147** | **1:500** |
| **MDA5** | **Anti-goat IgG Rhodamine Red-X** | **Donkey** | **Jackson Immuno Research, Grove, PA** | **705-296-147** | **1:200** |
