# Supplementary material for: Bi-glandular and persistent enterovirus infection and distinct changes of the pancreas in slowly progressive type 1 diabetes mellitus
Source: Sci Rep. 2023 Apr 28;13:6977. doi: 10.1038/s41598-023-33011-7 (PMC10147722; doi:10.1038/s41598-023-33011-7)
Supplement: Supplementary file 2 — Supplementary Figures. [file 41598_2023_33011_MOESM2_ESM.pdf]

Supplementary Fig. S1.

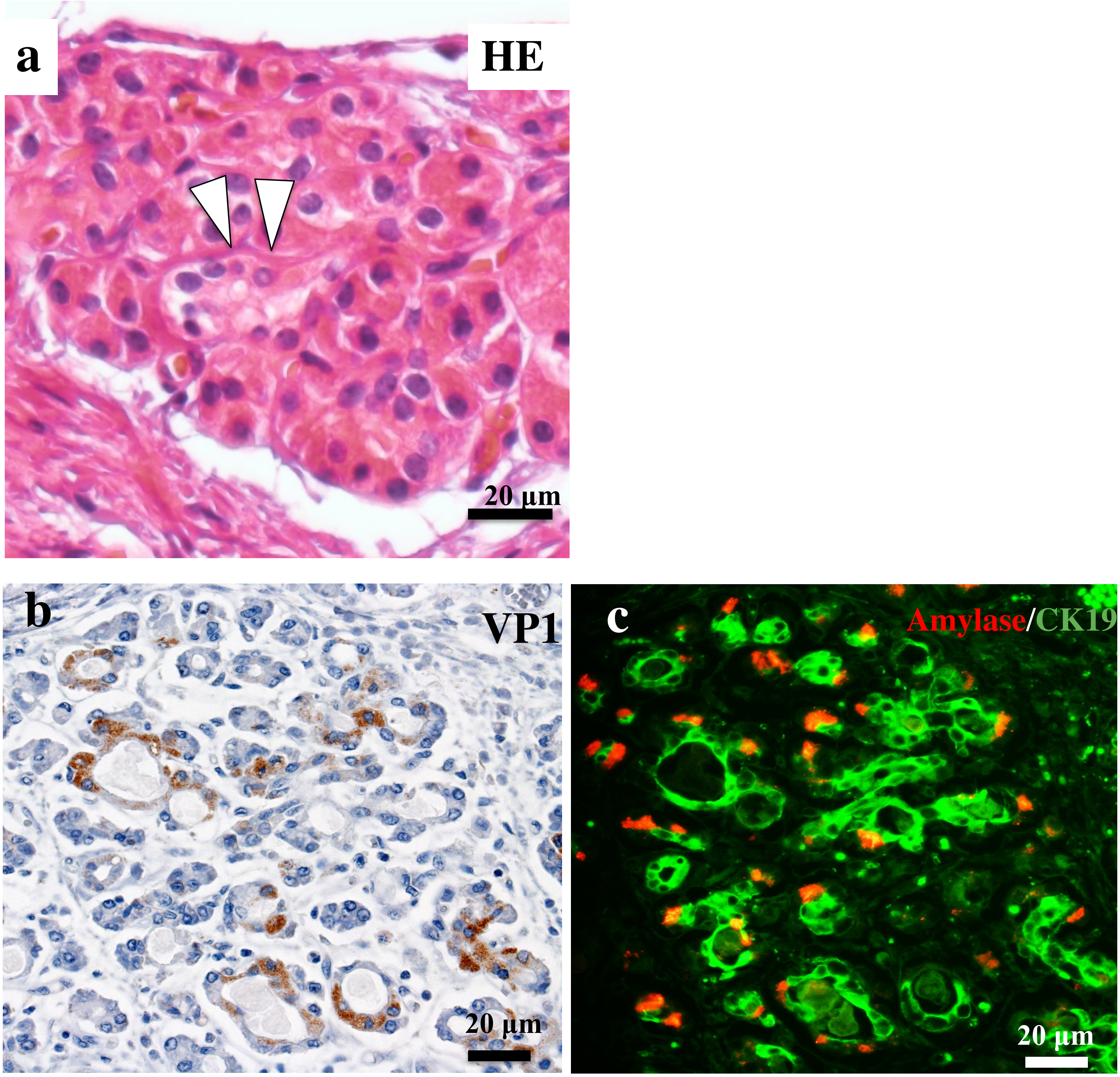

Supplementary Fig. S1. Characterization of acinar cells changed to acinar-to-ductal metaplasia (ADM) in the pancreas of SPIDDM

a. HE staining for consecutive section of the VP1-positive ring-like cells (Fig 1c) shows loss of eosinophilic feature, representing presence of zymogen granules (arrow heads) in a recent-onset SPIDDM (Case SP10).

b, c: Triple immune staining for VP1, cytokeratin 19 (CK19) and amylase showed some flat ring-like arrangement reminiscent for ADM cells (b, brown), exhibited CK19-positive (c, green) and amylase-positive (red), representing features of ADM in a long duration of SPIDDM (Case SP8).

Supplementary Fig. S2.

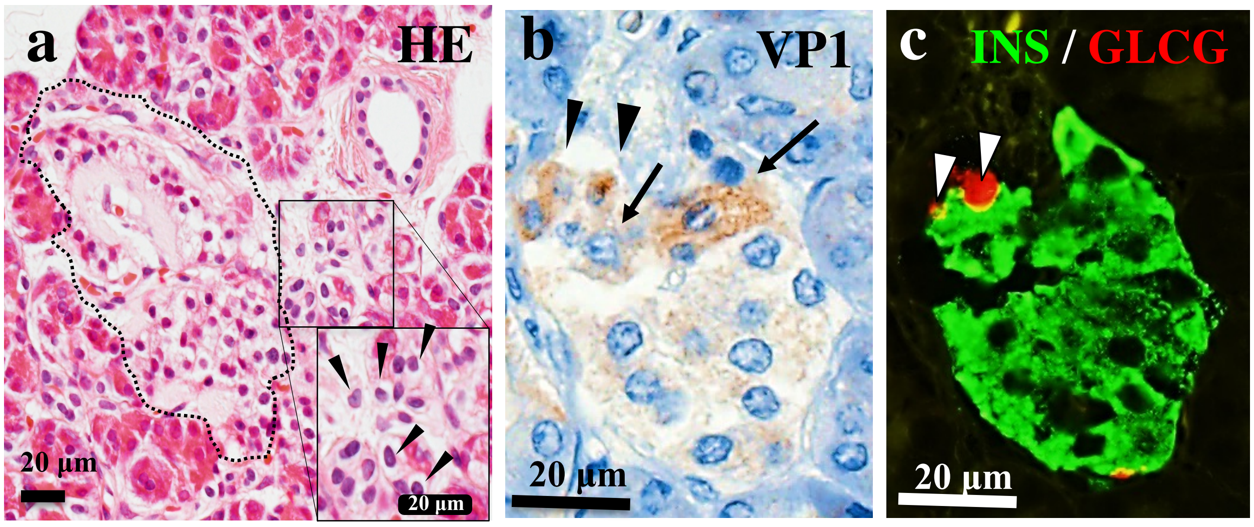

Supplementary Fig. S2. Partial de-granulation in acinar cells, and enterovirus (EV) tropism to islets cells in recent-onset SPIDDM.
a: Serial section with the section presented by Fig. 1b, in which EV-VP1 was positive in rectangular area, stained by Hematoxylin and Eosin (Case SP10). Magnified view (inset) shows some acinar cells composing acini, which lost eosinophilic character (arrowheads) representing partial degranulation of zymogen granules, sign of beginning of acinar-to-ductal metaplasia.
b, c: Triple immunostaining for VP1 (b, brown), insulin (c, green) and glucagon (c, red) in SPIDDM (Case SP10) shows that both beta cell (arrows), alpha-cell (arrowheads) are positive for VP1.

Supplementary Fig. S3.

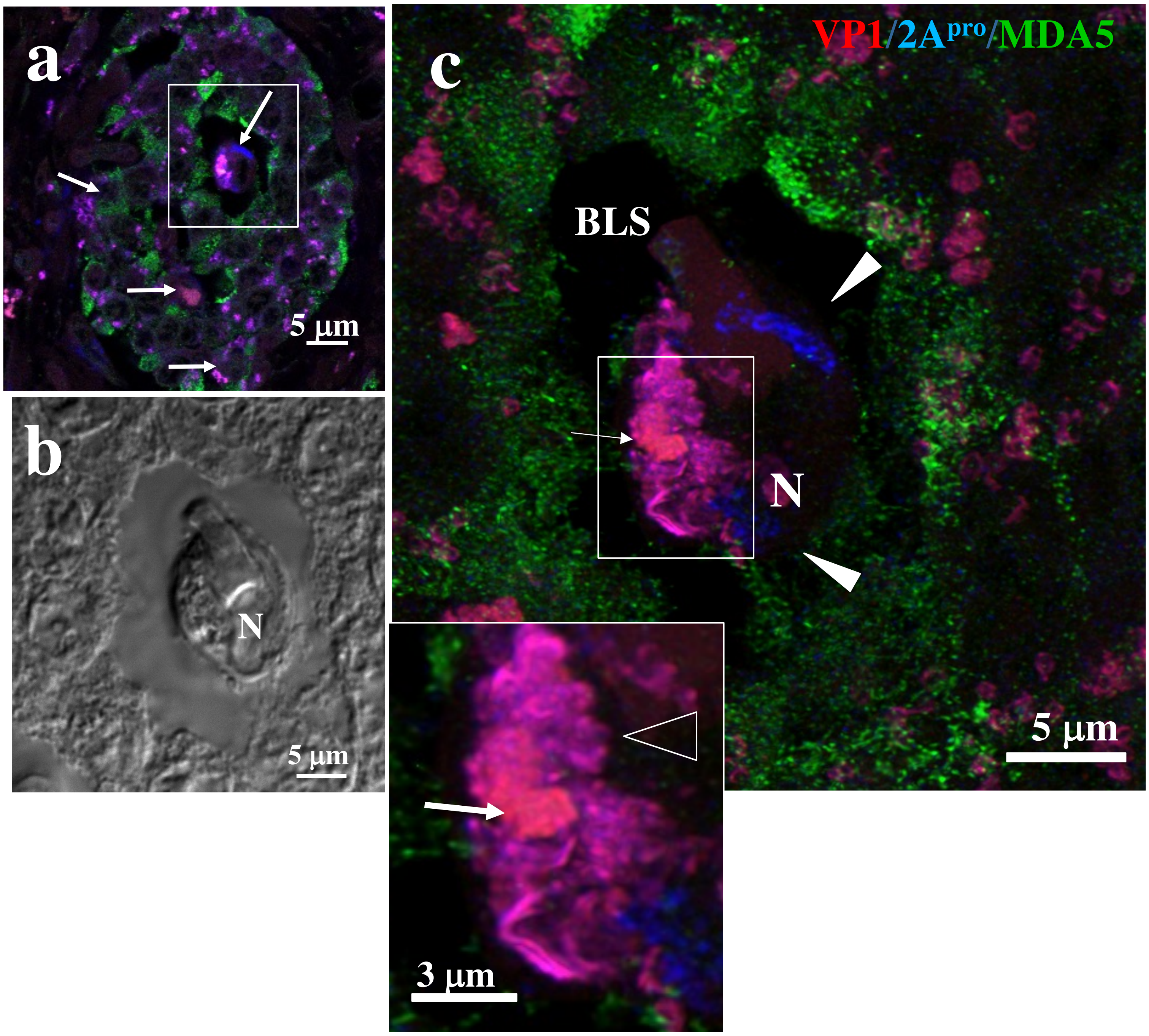

Supplementary Fig. S3.

a: Triple immunostaining for VP1, 2A^{pro} and MDA5 cells in the islet of SPIDDM (case SP-3). MDA5 expression was lost around VP1/2A^{pro}-positive islets cells (arrows) named as blank spots (BLS). **b**: Phase contrast view of the rectangular part in **(a). c:** At the center of BLS, VP1 (red, arrow), 2A^{pro} (blue, arrowheads) locate a cell around nucleus (N). VP1 (red) and 2A^{pro} (blue), which make a conglomerate, shows apparently magenta color (arrowhead, inset). This image was taken by TauSense, STELLARIS 8, Leica Microsystems, Germany.

Supplementary Fig. S4.

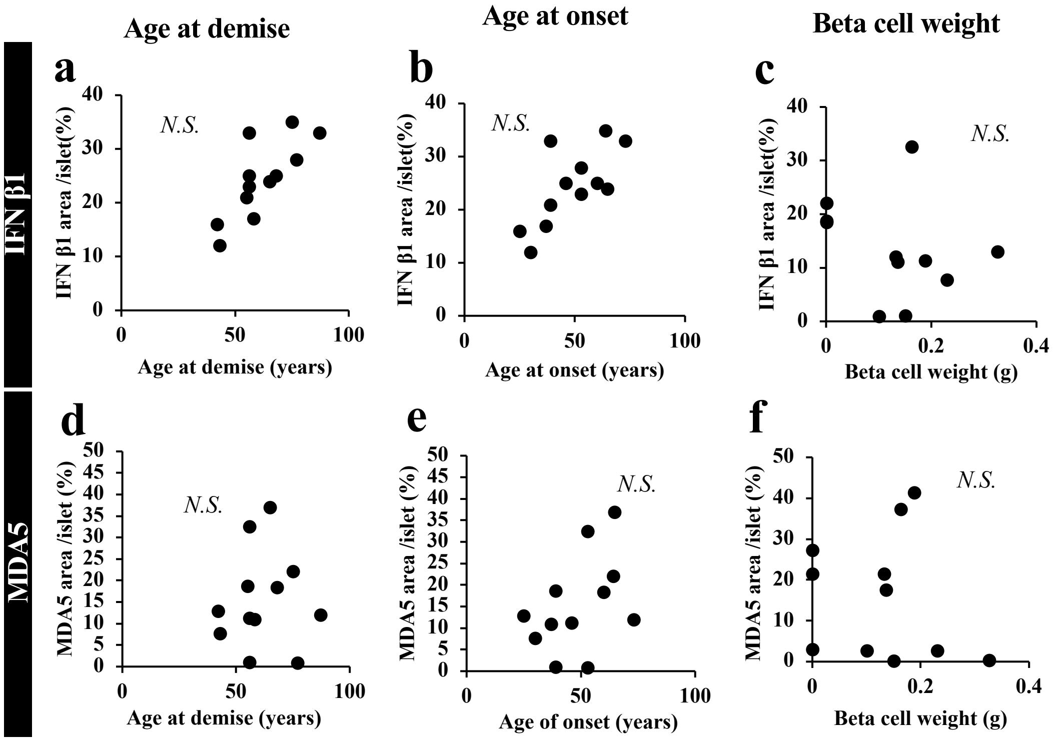

Supplemental Figure 4. No association was found between IFN beta1area and MDA5 area in the islets and age at demise (a, d), age at onset of diabetes (b, e) and estimated beta cell weight (c, f) of SPIDDM pancreas.

Supplementary Fig. S5.

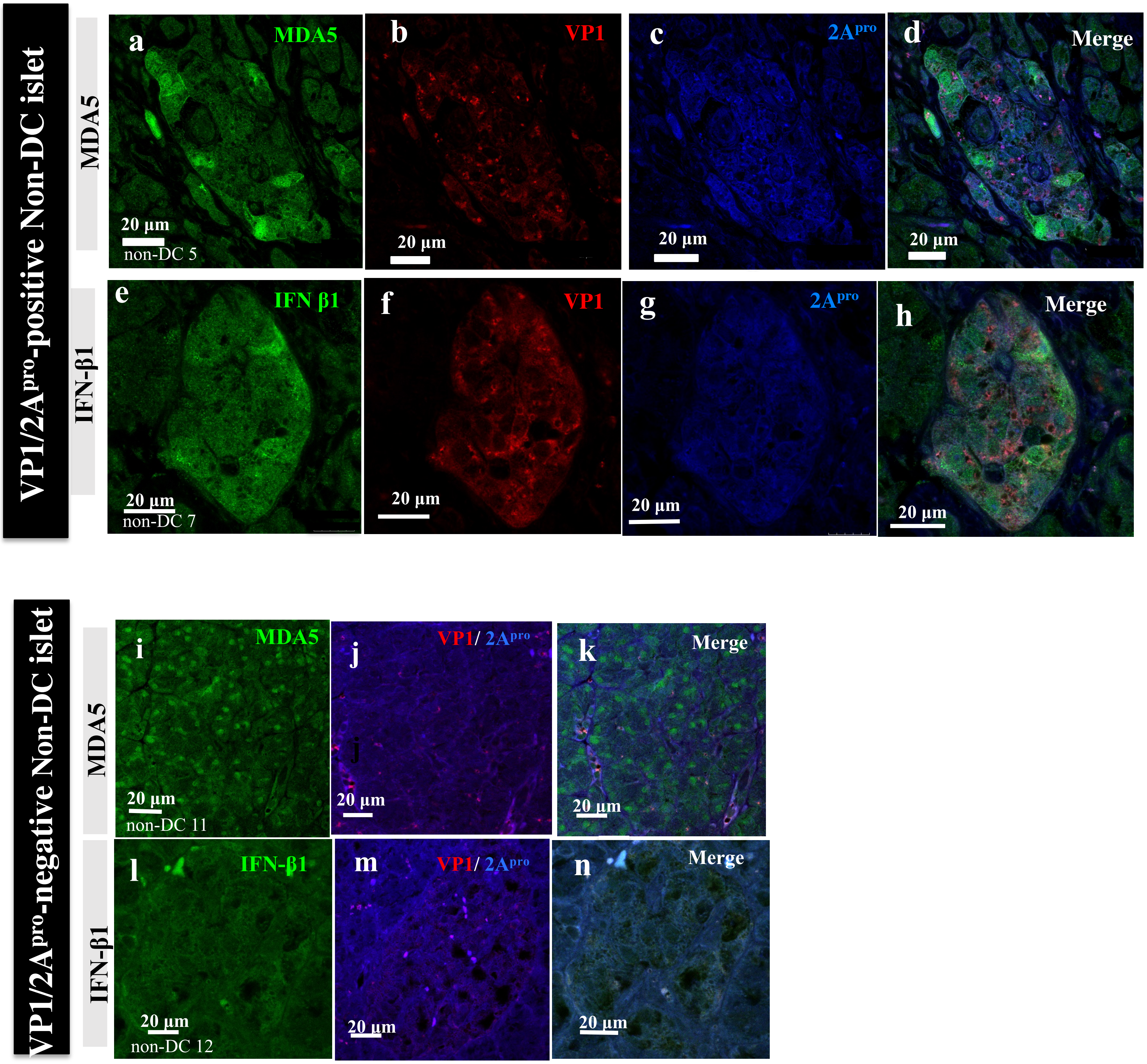

Supplementary Fig S5. EV-VP1 (VP1), 2A protease (2A^{pro}), MDA5 and IFN-β1 in VP1-positive and -negative non-diabetic control (non-DC) islets.
a-h: VP1- and 2A^{pro}- positive non-DC islets (b, c and f, g) showed expression of MDA5 (a), IFN-β1 (e). **d, h:** merged image of (a)-(c) and (e)-(g), respectively.
i-n: VP1- and 2A^{pro}- negative non-DC islets stained for MDA5 (i), IFN β1(l) showed absence of these molecules. Scale bar, 20 µm

Supplementary Fig. S6.

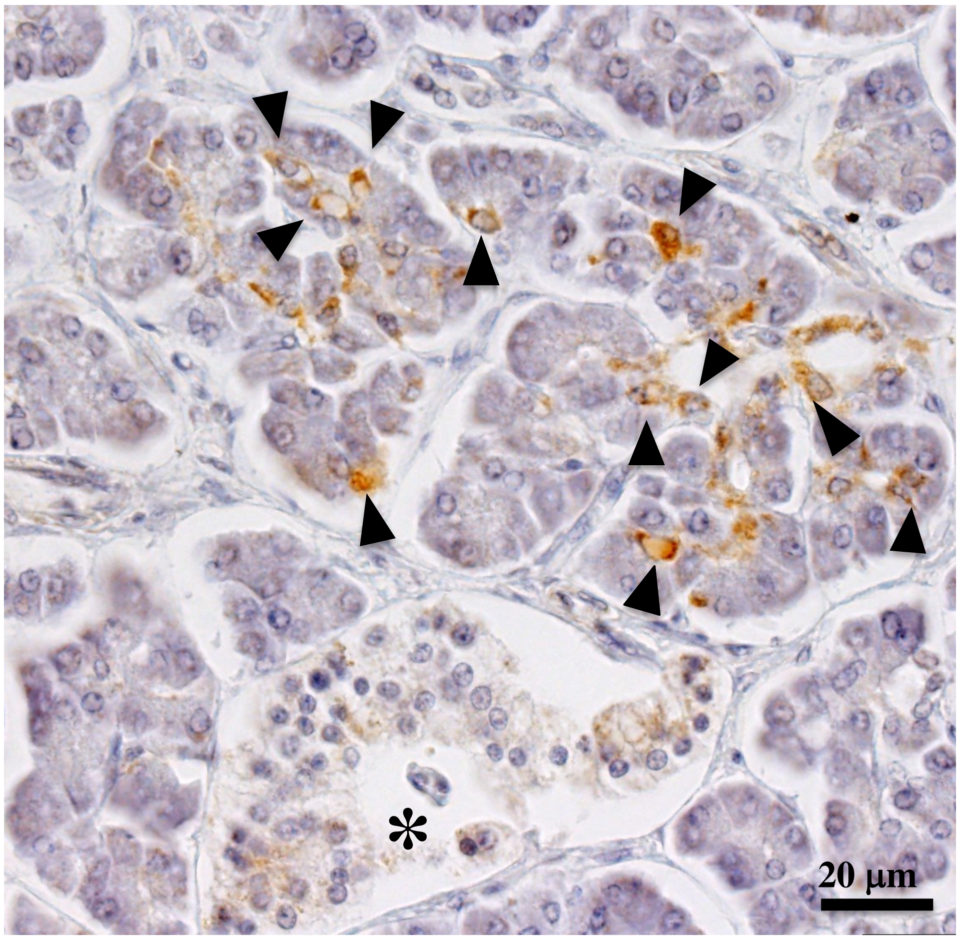

Supplementary Fig. S6.. Enterovirus VP1-positive and spindle-shaped centroacinar cells (brown, arrowheads) scattered in some acini aside VP1-positive islet (asterisk) of a Non-DC (Case non-DC 17).

Supplementary Fig. S7.

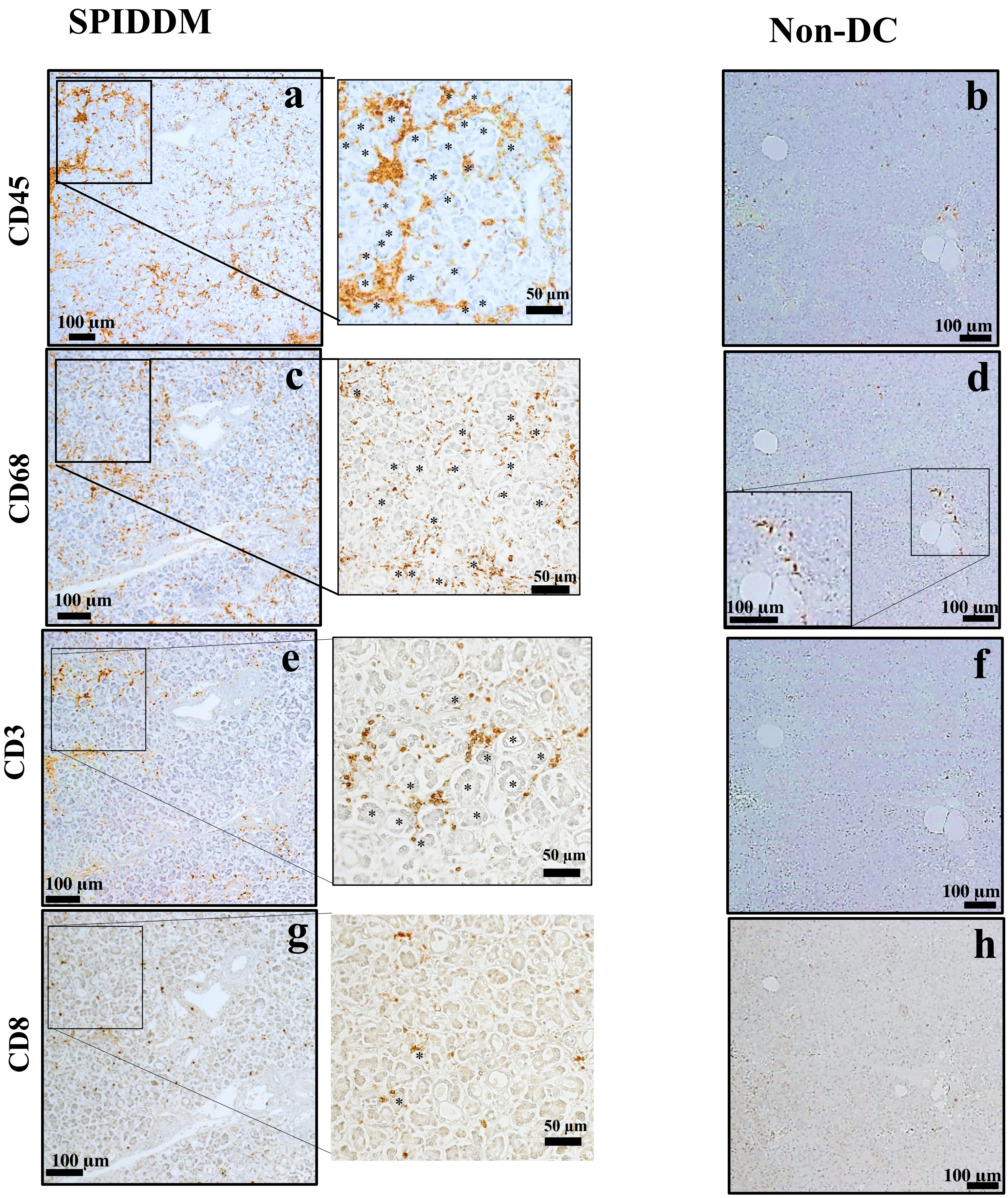

Supplemental Figure 7.

Increased numbers of various phenotypes of immune cells in the SPIDDM exocrine pancreas. Representative examples of the exocrine pancreas in non-PanIN lobe of SPIDDM (SP6, left panel) and a Non-diabetic control (non-DC8, right panel) stained with CD45, CD68, CD3, CD8, CD4, CD20, CD11c, and enterovirus VP1 (VP1), respectively. **a:** CD45^{+} cells infiltrate around exocrine acinar cells (left inset) and islets (dotted circle, right inset).

(legend continued on next page)

Supplementary Fig. S7. *(continued)*

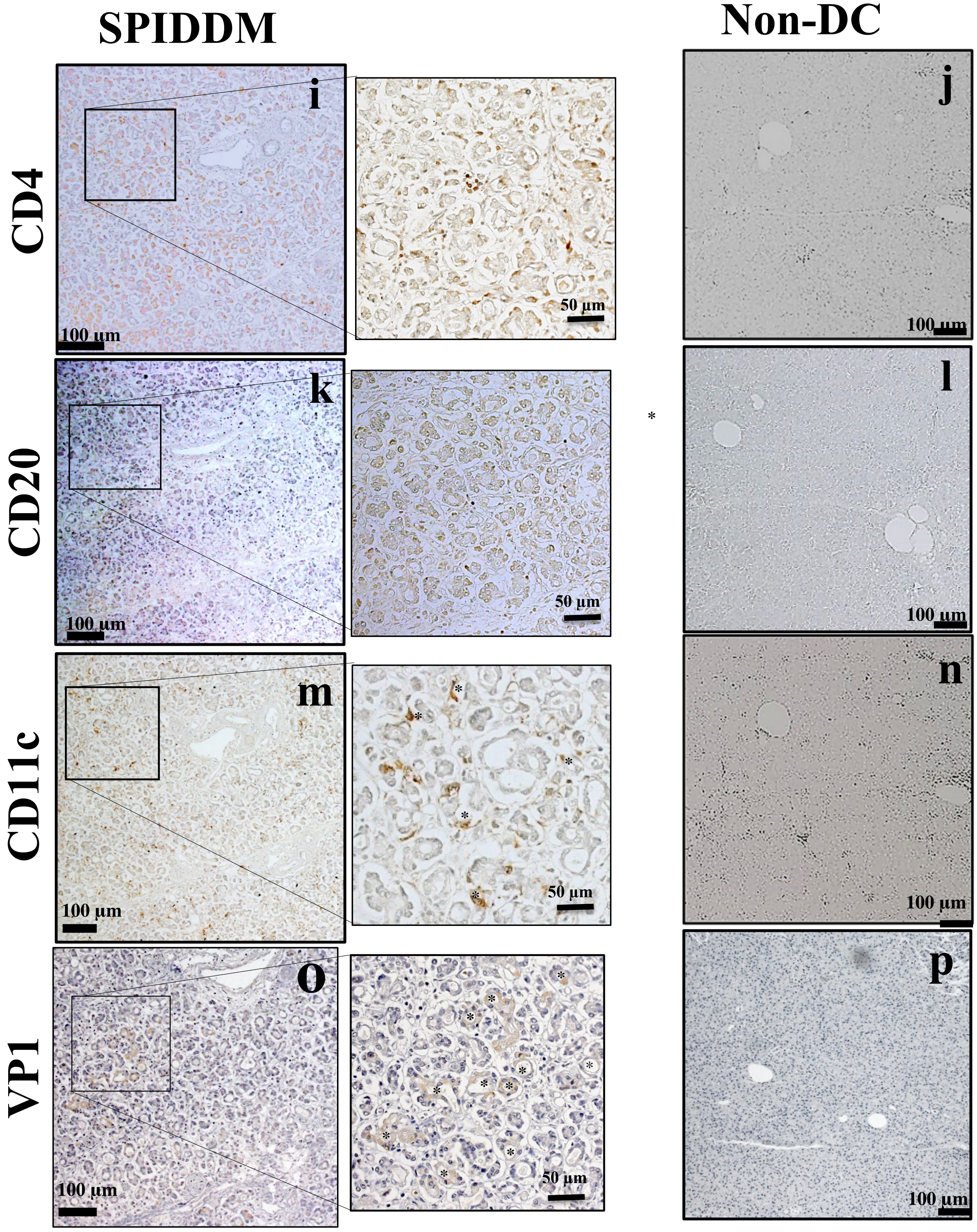

CD68+ macrophages reside around exocrine cells changed to acinar-to-ductal metaplasia (ADM, asterisks) in SPIDDM (**c**), and resident macrophages are quiescent under steady state in non-DC (**d**). CD3+ and CD8+ cells infiltrate around acinar cells with ADM (asterisks), which are flat acinar cells arranged like a ring (**e, g**). CD4+ cells and CD20+ cells are distributed homogeneously (**i, k**). CD11c+ cells surround ADM-positive acinar cells (**m**). VP1-positive acinar cells changed to ADM (inset, asterisks) are observed (**o**). **b, d, f, h, j, l, n, p**: Non-diabetic control. Scale bar, 100 µm unless otherwise noted.

Supplementary Fig. S8.

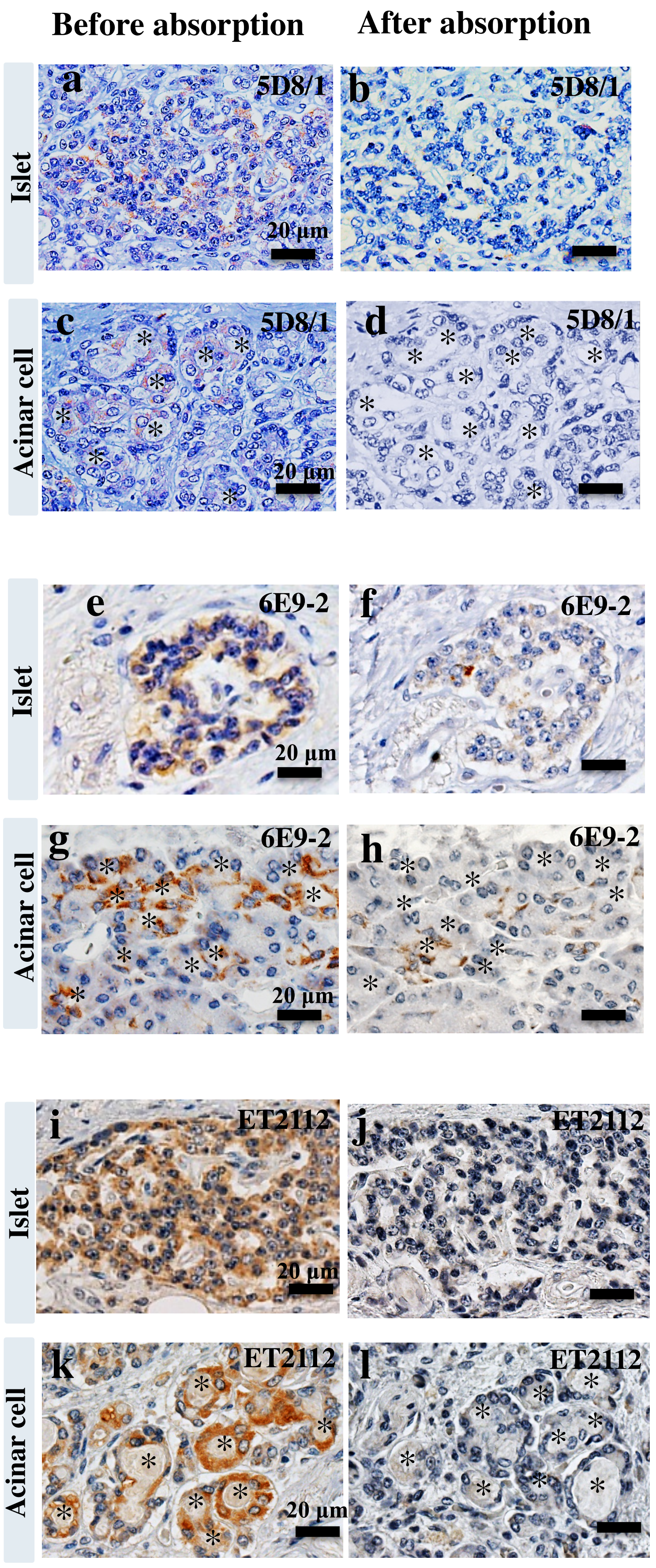

Supplementary Fig. S8. Absorption test for 5D8/1 (a-d) and 6E9-2 (e-h) by synthetic enterovirus VP1 protein and antiserum ET2112 (i-l) by synthetic enterovirus 2A protease (2A^{pro}) stained by the immunohistochemical method. Immunoreactivity of antisera for VP1 (5D8/1 and 6E9-2) and 2A^{pro} (ET2112) in the islets and pancreatic acinar cells changed to acinar-to-ductal cell metaplasia (ADM) in SPIDDM is completely absorbed in the islets and in exocrine acinar cells by immune peptides. Scale bar, 20 µm.

Supplementary Fig. S9

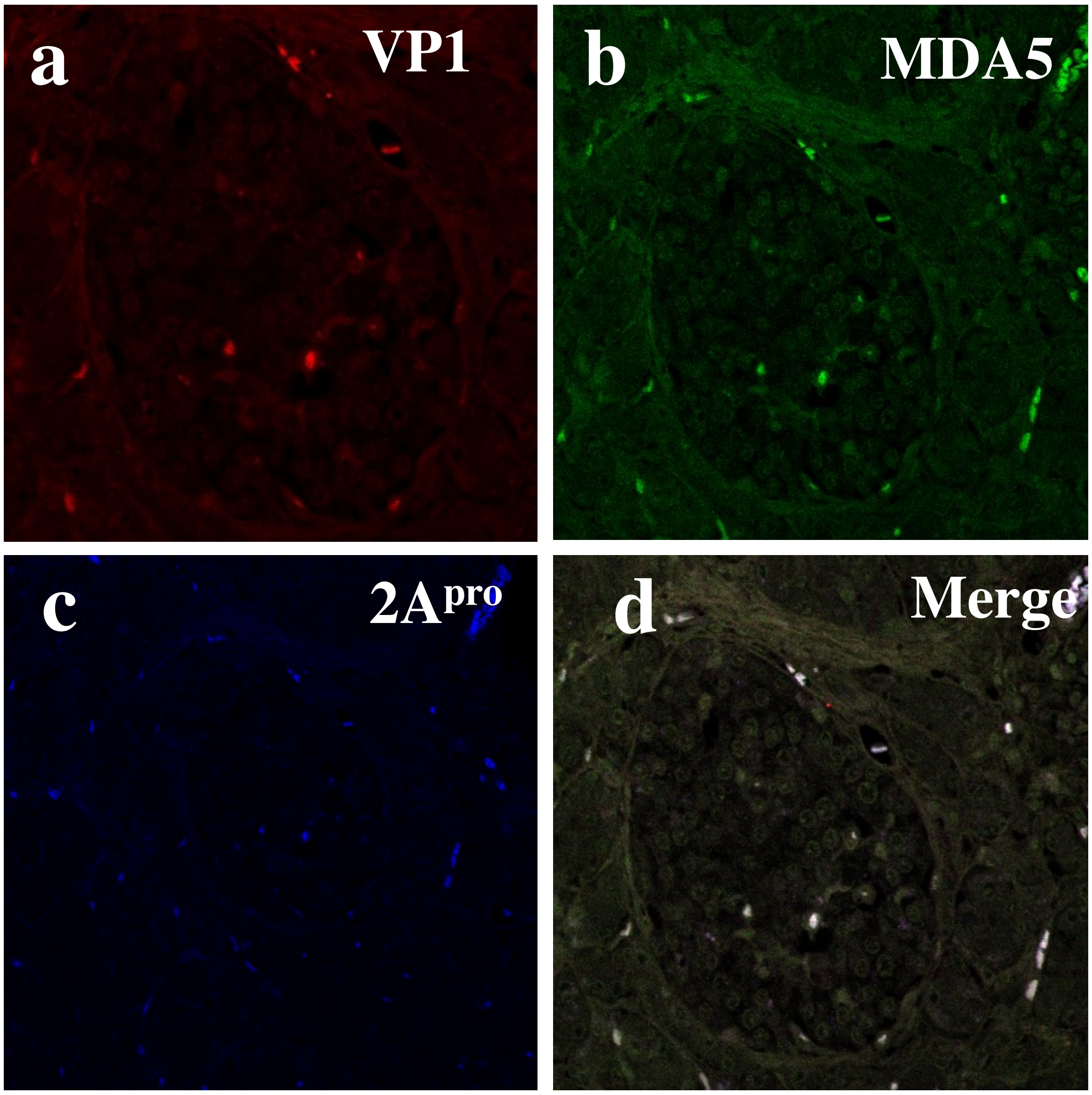

Supplementary Fig. S9. Negative control study eliminating primary antibodies for VP1, MDA5 and 2A^{pro} (a-c). Weak autofluorescence of nucleus of islet cells and pancreatic fibrous tissue were observed (d). Confocal microscopy.

Supplementary Fig. S10.

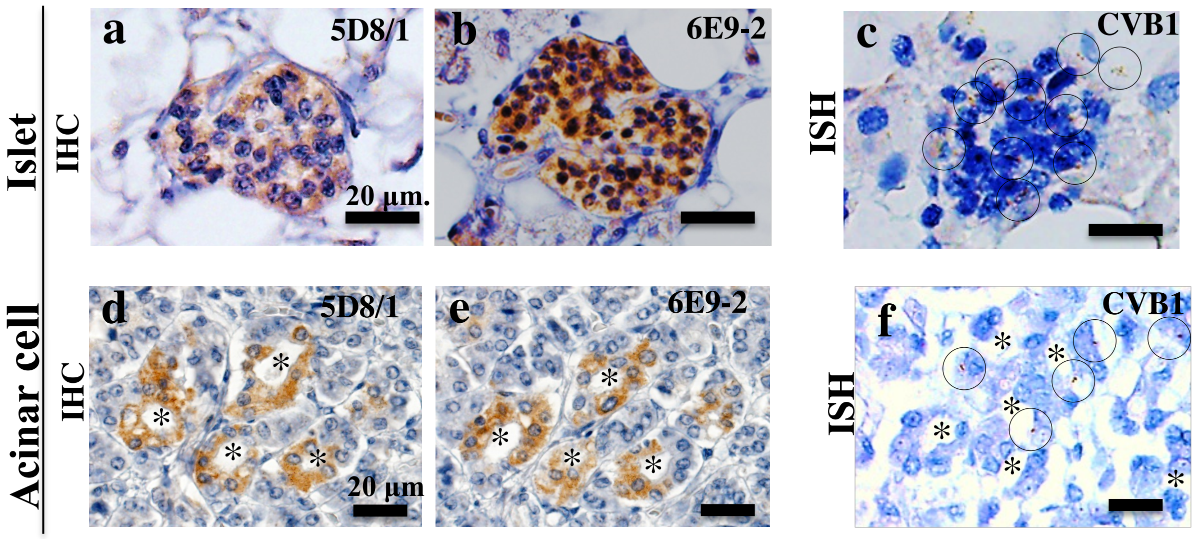

Supplementary Fig. S10. Staining of consecutive pancreatic sections of SPIDDM for enterovirus (EV) VP1 by immunohistochemistry (IHC) using 5D8/1 and 6E9-2 antibodies and EV-RNA by *in situ* hybridization (ISH)

a-c Serial sections of islets stained for VP1 by IHC using antisera 5D8 (a, brown) and 6E9-2 (b, brown) and EV-RNA detected by in situ hybridization (ISH) (c, brown dots pointed by circles) concorded well in the islet.

d-f Serial sections of exocrine pancreas stained for VP1 by IHC using antisera 5D8 (d) and 6E9-2 (e) and EV-RNA detected by ISH (f, brown dots pointed by circles). VP1 was stained by IHC using two antisera in the acinar cells changed to acinar-to-ductal metaplasia (asterisks) and location of VP1 concorded well in acinar cells by brown dots and shown by circles.

Supplementary Fig. S11.

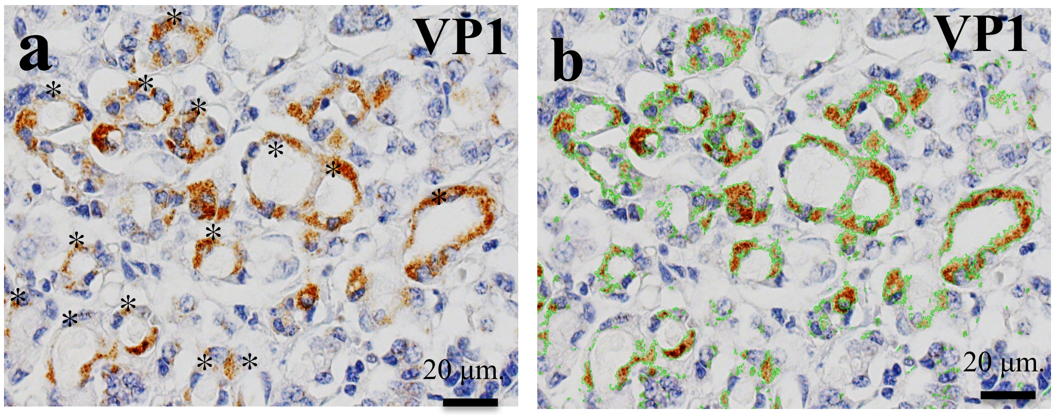

Supplementary Fig. S11. Capture of images of ADM cells positive for VP1 in the pancreas of SPIDDM

a, b: ADM changed acinar cells (a, brown), which were positive for EV-VP1 were well captured and well segmented by computer software (cellSens Dimension, Ver.1. 16, Olympus, Tokyo, Japan) (b, green).
